# Functional Vascular Smooth Muscle-like Cells Derived from Adult Mouse Uterine Mesothelial Cells

**DOI:** 10.1371/journal.pone.0055181

**Published:** 2013-02-06

**Authors:** Christian Claude Lachaud, Daniela Pezzolla, Alejandro Domínguez-Rodríguez, Tarik Smani, Bernat Soria, Abdelkrim Hmadcha

**Affiliations:** 1 Andalusian Center for Molecular Biology and Regenerative Medicine (CABIMER), Sevilla, Spain; 2 Instituto de Biomedicina de Sevilla/Fisiopatología Cardiovascular, Sevilla, Spain; 3 CIBER de Diabetes y Enfermedades Metabólicas asociadas (CIBERDEM), Barcelona, Spain; William Harvey Research Institute, Barts and The London School of Medicine and Dentistry, Queen Mary University of London, United Kingdom

## Abstract

In mammalian visceral organs, vascular smooth muscle cells (VSMCs) originate from an epithelial-to-mesenchymal transition (EMT) of embryonic mesothelial cells (MCs). The ability of adult MCs to recapitulate EMT and to acquire smooth muscle (SM) markers upon provasculogenic culture suggested they might retain embryonic vasculogenic differentiation potential. However, it remains unknown whether adult MCs-derived SM-like cells may acquire specific vascular SM lineage markers and the functionality of differentiated contractile VSMCs. Here, we describe how a gentle trypsinization of adult mouse uterine cords could selectively detach their outermost uterine mesothelial layer cells. As other MCs; uterine MCs (UtMCs) uniformly expressed the epithelial markers β-catenin, ZO-1, E-cadherin, CD54, CD29, and CK18. When cultured in a modified SM differentiation media (SMDM) UtMCs initiated a loss of epithelial characteristics and gained markers expression of EMT (Twist, Snail, and Slug), stem and progenitor (Nanog, Sox2, C-kit, Gata-4, Isl-1, and nestin), SM (α-SMA, calponin, caldesmon, SM22α, desmin, SM-MHC, and smoothelin-B) and cardiac (BMP2, BMP4, ACTC1, sACTN, cTnI, cTnT, ANF, Cx43, and MLC2a). UtMCs repeatedly subcultured in SMDM acquired differentiated VSM-like characteristics and expressed smoothelin-B in the typical stress-fiber pattern expression of contractile VSMCs. Relevantly, UtMCs-derived VSM-like cells could generate *“mechanical force”* to compact collagen lattices and displayed in diverse degree voltage (K^+^) and receptor (endothelin-1, oxytocin, norepinephrine, carbachol and vasopressin)-induced [Ca^2+^]*_i_* rises and contraction. Thus, we show for the first time that UtMCs could recapitulate in vitro differentiative events of early cardiovascular differentiation and transdifferentiate in cells exhibiting molecular and functional characteristics of VSMCs.

## Introduction

Mesothelial cells (MCs) are squamous epitheloid cells lining pleural, pericardial and peritoneal body cavities and the visceral organs housed within. The main functions of MCs are to provide a protective barrier and lubricating surface for the optimal sliding of organs inside body cavities. Although MCs are derived from the mesoderm, they rather resemble simple epithelial cells, and as such, they express epithelial markers and can undergo an epithelial-to-mesenchymal transition (EMT), a transdifferentiation mechanism inducing their loss of *epithelial characteristics (i.e, disruption of junctional complexes, cell-cell polarity and cell-matrix adhesions) and the acquisition of a migratory mesenchymal phenotype* [1], [2].

Initial evidences of mesothelial *EMT arose from developmental studies conducted* in the embryonic avian heart, where proepicardial-derived MCs were found to undergo EMT and migrate into submesothelial layers where they differentiate into coronary VSMCs, interstitial fibroblasts and possibly endothelial cells [3], [4]. This unusual vasculogenic mechanism set a breakthrough in previous concept of embryonic blood vessel development thought to be mediated exclusively by endothelial tubes inducing proximal mesodermal progenitors to differentiate into VSMCs and pericytes [5]. Mouse mesothelial lineage-tracing studies further confirmed a similar conversion of embryonic MCs into stromal and vasculogenic mesenchymal phenotypes in the developing heart, gut, lung and liver [6]–[10].

Thus far, the occurrence of mesothelial EMT has not been reported in healthy adults, even if pathophysiological mesothelial EMT often develops over time in several fibrotic processes (i.e, lung, liver and kidney fibrosis) [2] or in the peritoneum of patients who are on continuous ambulatory peritoneal dialysis [11], [12]. Among the known inducers of peritoneal fibrosis, the profibrotic factor TGF-β1 has emerged as a master inducer of peritoneal MCs EMT and fibrosis [13]. Cumulating number of works indicates that healthy adults MCs retain the capability to recapitulate an EMT and to acquire components of the SMCs contractile machinery (α-SMA, SM-myosin, α-tropomyosin, calponin and SM22α) upon provasculogenic culture (i.e, culture media containing fetal bovine serum or purified recombinant provasculogenic and morphogenic growth factors such as TGF-β1, PDGF-BB, bFGF and EGF [14]–[19]. Such findings led to the suggestion that adult MCs might retain vasculogenic differentiative mechanisms [14]–[16]. Indeed, it was found that adult MCs-derived SM-like cells can secrete SM-related matrix proteins (i.e, fibronectin and collagen type I) and proteolytic enzymes (i.e, metalloproteinases 2 and 9) which are required for SMCs migration [12], [19]. In addition, adult MCs-derived SM-like cells exhibit signaling through Smad 3 and activation of the phosphatidylinositol 3 kinase (PI3K)/Akt pathway [20], [21], which are two key signaling events for the early SM differentiation of Embryonic Stem Cells (ESCs) [22].

Other works however suggested that the α-SMA^+^ SM-like cells produced by EMT of adult MCs might represent myofibroblasts [18], [23]. The close phenotypic similarities between SMCs and myofibroblasts may largely explain the controversial lineage identity of the adult MCs-derived SM-like cells. Indeed, the different contractile markers (α-SMA, SM-myosin, α-tropomyosin, calponin and SM22α) reported to be expressed in the different adult MCs-derived SM-like cells populations may not been completely specific to the SM lineage as evidenced by the report of their detection in myofibroblastic tissues and cultured myofibroblasts [24]–[27]. In vitro, it may be inclusively more difficult to distinguish SMCs from myofibroblasts, since serum induces SMCs to dedifferentiate towards a proliferative synthetic phenotype displaying reduced contractile markers and contractile mechanisms [28]–[30], a phenotype that is likely to be closer to that of serum-cultured myofibroblasts.

Other component of the SMCs contractile machinery such as desmin, h-caldesmon and smoothelin may however not be expressed in the myofibroblastic lineage [26], [31]–[33]. Of particular relevance, alternative splicing of the smoothelin gene generates at least two isoforms, being the short smoothelin-A isoform (59 kDa) specifically expressed into contractile visceral SMCs, whereas the long smoothelin-B isoform (110 kDa) is thought to be expressed only in adult contractile VSMCs [34], [35]. Therefore, the detection of smoothelin-B, together with other specific components of the SMCs contractile machinery might represent key markers to definitively clarify whether adult MCs can truly transdifferentiate into contractile VSMCs. Furthermore, it remains to evaluate in depth the capacity of adult MCs-derived SM-like cells to acquire VSMCs functionality (i.e, acquisition of vasoactive agonists-induced calcium and contractile responses).

Herein, we show that a gentle trypsinization of adult mouse uterine cords can efficiently and reliably detach from their surface a highly enriched population of UtMCs, that when cultured in a modified SM differentiation media (SMDM) could massively undergo EMT and gain expression of stem, progenitor and cardiovascular developmental markers. Their prolonged subculture in SMDM improved their maturation and acquisition of molecular and functional characteristics of differentiated VSMCs.

## Materials and Methods

### Isolation and Culture of UtMCs

Uterine cords were isolated from non-pregnant OF1 mice (6–20 weeks old). Mice were killed by cervical dislocation; the Animal Experimentation and Ethics Committee of CABIMER (CEEA-CABIMER) approved the procedure (permit number 19-2010). Uterine cords were isolated and incubated in 5 ml of phosphate-buffered saline solution (PBS) containing 2% bovine serum albumin (BSA) and 0.25% trypsin (Gibco, Paisley, UK) at 37°C for 30 minutes. Trypsin released UtMCs were centrifuged (500 g×5 min) and numbers of viable UtMCs were counted with the trypan blue exclusion assay.

According to the work of data previously reported by Ahmed et al. [50], we formulated a modified SM differentiation media (SMDM) to induce the vasculogenic differentiation of UtMCs. SMDM was formulated from a basal media (DMEM GlutaMax™ high glucose (4.5 g/L) media (Gibco) containing 10% heat inactivated fetal bovine serum (FBS, Hyclone), antibiotics (penicillin, 100,000 units/l and streptomycin, 50 mg/l; Gibco) and 100 µM β-mercaptoethanol (Gibco)) supplemented with 20 ng/ml of mouse recombinant EGF (PeproTech, London, UK) and 1 ng/ml hydrocortisone (Sigma Aldrich, Saint Louis, MO, USA).

UtMCs were plated (2.10^5^ cells/2 ml SMDM/well) into 6-wells tissue culture dishes (140685, Nunclon™ Δ Surface, Nunc, 140685) and expanded for 5 days in an incubator (5% CO_2_, 37°C). Media was changed every 2 days. UtMCs were then harvested with 0.05% trypsin and subcultured (20000 cells/cm^2^) in 10 ml SMDM in 100 mm Petri dishes (172958, Nunclon™ Δ Surface, Nunc) for other 5 days. A second subculture step of 5 days was identically performed to obtain UtMCs-derived VSM-like cells.

### Isolation and Culture of Primary Rat Aortic VSMCs

Aortas were isolated from adult rats Wistar with fine scissors, washed in PBS, and incubated for 10 minutes in a 0.25% trypsin solution containing 2% BSA to detach perivascular and endothelial cells. Aortas were then cut into small pieces and partially digested during 10 minutes in a 2 mg/ml collagenase type-IA solution to obtain fragments of medial smooth muscle (SM) layer. SM fragments were then transferred to a 4-wells dish (Nunc Multidishes, Nunc, 176740) containing 400 µl of BM-*β* Me. Media was partially changed after 24 hours. On day 2, once fragments had firmly adhered to the substrate, the volume of media was completed to 1 ml. Migrating VSMCs were observable at days 3–4 and cultured until they reached confluence and they were harvested with 0.05% trypsin. VSMCs were subcultured at 5000/cm^2^ for other 7 days in BM-*β*Me and finally used to generate multilayered primary VSMCs cultures for their use in contraction experiments.

### Culture of A7r5 VSMCs and MS1 Endothelial Cells

Rat aortic A7r5 VSMCs (CRL-1444, ATCC, Manassas, VA, USA) were expanded in basal media. MS1 mouse endothelial cells (CRL-2279, ATCC) were cultured in DMEM GlutaMaxTM low glucose (Gibco) +10% FBS and 1% P/S.

### Immunofluorescence

For immunofluorescence characterization of freshly isolated UtMCs, cells were plated for 6 hours in poly-L-lysine treated CultureWell™ Chambered Coverglass (C37005, Grace Bio-Labs, Molecular Probes) in SMDM. For longer periods, UtMCs were cultured in hydrophilic µ-Dish (45079, Ibidi GmbH, Germany). For cell-surface antigens, cells were fixed 20 minutes with 4% PFA and blocked with PBS containing 3% BSA. For intracellular antigens, cells were permeabilized with 0.5% Triton X-100 (T8787, Sigma Aldrich) or in −20°C cold methanol for 20 minutes depending on the antigen detected as detailed in Table S1. Fluorescence images were captured with an inverted fluorescence microscope Olympus IX71 equipped with the digital image processing softwares DPController and DPManager (Olympus. www.olympus.co.uk). Nuclei were counterstained with 1 µg/ml Hoechst 33342 (Sigma Aldrich). F-actin was stained with Alexa Fluor® 633 phalloidin antibody (Invitrogen). Primary and secondary antibodies used in this study are listed in Table S1 and S2, respectively.

### Whole-Mount Immunofluorescence of Uterine Cords

Intact and trypsinized uterine cords were fixed with 4% PFA and processed as described previously for immunofluorescence detection of surface and intracellular antigens. At the end of the immunolabelling procedure, uterine cords were mounted in sandwich between two glass coverslips and immunofluorescent images were taken with an inverted fluorescence microscope Olympus IX71.

### MetaMorph-Based Fluorescence Signals Quantification

Phenotypic changes undertaken by UtMCs during their culture in SMDM related to variations in markers expression were analyzed by quantifying the signals of immunofluorescence detection of E-cadherin, CK18, α-SMA and nestin using Meta Imaging Software MetaMorph Offline version 7.5.1.0 (MDS Analytical technologies, Sunnyvale, CA, USA). Analysis was performed using the ratio of the intensity values of Alexa 488 nm and Hoechst 33342 above background immunofluorescence emitted by the substrate and the data were expressed as arbitrary units (AU) and exported automatically from MetaMorph to Microsoft Excel program trough a summary log. The numeric results are presented as mean ± s.d. performed in 3 independent cultures.

### Flow Cytometry Quantification of Cell Surface and Intracellular Markers

Flow cytometry analysis was performed using a FACSCalibur Flow Cytometer (Becton, Dickson and company, USA). For surface markers, cells were fixed with 4% PFA and blocked with 3% BSA for 1 hour at 4°C. Aliquots of cells were then incubated at 4°C during 30 minutes with fluorescent-conjugated antibodies described in Table S3. Non-specific fluorescence was determined with isotype match conjugated antibodies. Parameters of capture were adjusted so that isotype controls were less than 0.5% positive.

Intracellular markers were labelled after permeabilizing cells with 0.5% triton-X for 1 hour at 4°C. Aliquots of cells were then incubated for 1 hour at 4°C with primary antibodies followed by their incubation with secondary antibodies for 30 minutes. Negative controls were cells incubated with secondary antibodies only.

### Measurements of Cytosolic Ca^2+^

Measurement of cytosolic Ca^2+^ [Ca^2+^]*_i_* concentrations was performed in UtMCs cultured onto small glass coverslips (25 mm^2^). UtMCs were loaded during 30 minutes with 2 µM fura-2AM in a Teflon chamber. Experiments were performed in a standard solution [2 mM Ca^2+^, 140 mM NaCl, 4.7 mM KCl, 1 mM MgCl_2_, 10 mM HEPES (pH = 7.4)]. Fluorescence was monitored using a Nikon TS-100 inverted microscope equipped with a 20X Super Fluor objective (0.75 NA) as described previously [67], [68]. Fluorescence images of 20 to 30 cells were recorded and analyzed with a digital fluorescence imaging system InCyt IM2 (Intracellular Imaging Inc, Imsol, UK) equipped with a light sensitive CCD camera (Cooke PixelFly, ASI, Eugene, OR, USA). Changes in [Ca^2+^]*_i_* concentrations are represented as the ratio of fura-2 fluorescence induced at an emission wavelength of 510 nm due to excitation at 340 nm and 380 nm (ratio = F_340_/F_380_). Ca^2+^ influx was determined from changes in fura-2 fluorescence after addition of a given vasoactive agonist. Δ ratio was calculated as the difference between the peak ratio response after contractile agonist was added and its level right before its addition.

### Detection of Functional Muscarinic Acetylcholine Receptors

The presence of functional muscarinic acetylcholine receptors (mAChRs) onto the SMDM cultured UtMCs, was determined by measuring their capacity to display significant rises of [Ca^2+^]*_i_* in response to carbachol, a potent agonist for muscarinic and nicotinic receptors. In a first set of experiments, UtMCs cultured for 5 days in SMDM (to induce their vasculogenic differentiation) or in a serum-free media (to retain their mesothelial phenotype) were loaded with fura-2AM and challenged against 1 mM carbachol and 5 µM of adenosine 5′-triphosphate (ATP, Sigma Aldrich). Serum-free media consisted in a DMEM low glucose media supplemented with 1% P/S and 1X B27 supplements. In other experiments, UtMCs that were cultured for 5 and 15 days in SMDM were preincubated with 1 mM atropine and challenged against 1 mM carbachol.

### Vasoactive Agonists and Antagonists

The vasoactive agonists used were; KCl (60128), carbachol (C4382), endothelin 1 (E7764), angiotensin II (A9525), [Arg^8^]-arginine vasopressin (V9879), oxytocin (O3251), norepinephrine (A9512), acetylcholine (A6625) and oxotremorine (O6876). Antagonists were; atropine (A0132), BQ-123 (B150) and BQ-788 (B157). All were from Sigma Aldrich (Saint Louis, Missouri, USA).

### Cells-collagen Gel Lattices Contraction Assay

Cells-collagen lattices were performed as described previously [48] with minor modifications. The contractile capacity of collagen lattices harbouring UtMCs-derived VSM-like cells (UtMCs cultured for 15 days in SMDM) was compared against that of collagen lattices containing freshly isolated UtMCs or MS1 endothelial cells. Cell suspensions were adjusted to 2.10^6^ cells/ml in cold 2X α-MEM (Sigma Aldrich) and adjusted to a proportion 1∶1 with a precooled 4.6 mg/ml rat tail collagen type I solution (BD Biosciences, 354236). Cells-collagen mixtures (750 µl) were poured into 12-well culture plates and allowed to polymerize at 37°C for 30 minutes. Lattices were then mechanically released from the well by gentle addition of 1 ml of SMDM and cultured for 48 hours. Media added to MS1 endothelial cells-collagen lattices was the same as described above for their culture. Medias were renewed after 24 hours of culture. Digital photographs of collagen gel lattices were taken at 0, 24 and 48 hours of their release and used to quantify lattices areas with MetaMorph Software.

### Generation of Multilayered UtMCs-derived VSM-like Cells Cultures

UtMCs-derived VSM-like cells (UtMCs cultured 15 days in SMDM) were harvested with 0.05% trypsin. A total of 2.10^6^ cells in 5 ml SMDM was transferred into a 6 Wells Ultra Low Attachment Plate (3471, Costar) and incubated for 24 hours to force their aggregation in spheroids of average 200–300 µm diameter. An average of 50 spheroids into 1.3 ml SMDM was distributed per well of 4-well culture plates (Nunc). Spheroids were then pooled to the centre of the well and cultured for other 24 hours to achieve their partial spreading onto the substrate and their subsequent reorganization into multilayered UtMCs-derived VSM-like cells cultures.

### Vasoactive Agonists-induced Contraction of Multilayered UtMCs-derived VSM-like Cells Cultures

The capacity of multilayered UtMCs-derived VSM-like cells cultures to contract in response to several vasoactive agonists was evaluated by time lapse image recording under an inverted microscope Olympus IX71 equipped with a prewarmed working area (±37°C). A volume of 100 µl of media was removed from cultures and used to resuspend vasoactive agonists at 10X. Phase contrast images were taken each 20 seconds for a total duration of 20 minutes. Premixed vasoactive agonists were added to cultures after 5 minutes of phase contrast image recording. Movies were produced from the sequence of phase contrast images recorded. Intensity of contractile responses against a given vasoactive agonist was determined by comparison to a lack of contraction (additions of media alone) and to a strong contractile response that was recorded in multilayered primary aortic VSMCs cultures incubated with 50 nM endothelin-1.

### Reverse Transcription-Polymerase Chain Reaction

Total RNA content was extracted with Trizol reagent (Invitrogen, Carlsbad, CA, USA), and total RNA (1 µg) was reverse-transcribed into cDNA by MMLV reverse transcriptase (Promega). RT-PCR was carried out using the BIOTAQ DNA polymerase (BIOLINE, London, UK). Reactions containing specific primers were incubated at indicated annealing temperature (Table S4) and subjected to 35 cycles of amplification. The PCR reaction products were separated by electrophoresis in 2% agarose gels containing Gel Red (Biotium, Hayward, CA, USA).

### Western Blotting

Protein lysates were subjected to electrophoresis separation in 8–10% SDS-PAGE and transferred to a polyvinylidene difluoride membrane (Hybond-P, Amersham, Buckinghamshire, UK). Membranes were blocked in Tris-buffered saline with 2% BSA and 0.2% Tween 20. Blots were incubated overnight with primary antibodies (Table S1) and immunoreactive bands were detected by horseradish peroxidise-conjugated secondary antibodies (Table S2) followed by ECL Plus detection system (Amersham).

### Statistical Analysis

Values are presented as mean ± s.d. Statistical significance was calculated by using an unpaired Student’s t-test, *p*<0.05 was considered significantly different.

## Results

### Immunophenotype Characterization of Trypsin Isolated Uterine Mesothelial Cells

**Figure 1:**
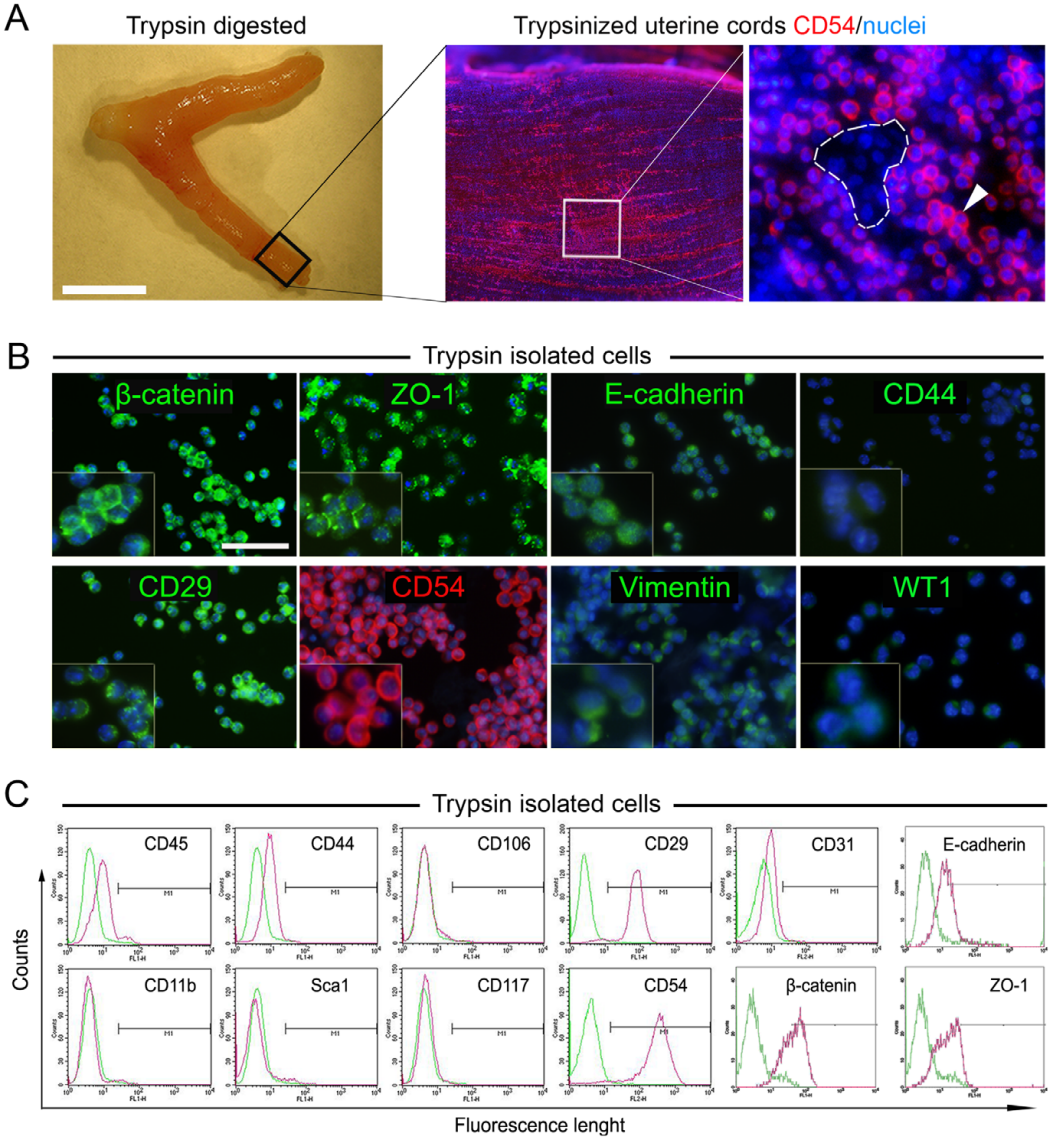
Efficient isolation of uterine mesothelial cells (UtMCs) by gentle trypsinization of uterine cords. (**A**) Left photograph shows representative aspect of uterine cords after their enzymatic digestion. Scale bar is 1 cm. Right panel shows CD54 immunofluorescence labelling of trypsinized uterine cords. Note that trypsinized uterine cords still retain CD54^bright^ UtMCs (spot) on their surface. Enlarged spot area shows CD54^bright^ UtMCs clearly distinguishable from CD54^low/negative^ submesothelial cells (dashed white area). (**B**) Trypsin isolated UtMCs widely immunoexpress β-catenin, ZO-1, E-cadherin, CD29, CD54 and vimentin, but are immunonegative against CD44 and α-SMA. Scale bar is 50 µm. (**C**) Flow cytometry analysis of trypsin isolated UtMCs indicating their expression (pink histograms) of CD54, CD29, β-catenin, E-cadherin and ZO-1 and lack of expression of CD45, CD11b, CD44, Sca1, CD106 and CD117. Green histograms are cells labelled with fluorescent-conjugated isotype-matched antibodies. (**A**, **B**) Nuclei are counterstained in blue with Hoechst 33342.

A gentle trypsinization of uterine cords resulted to be highly effective to selectively detach outermost uterine mesothelial cells that were termed UtMCs (Fig. 1). As for whole-mount immunofluorescence of intact uterine cords (Fig. 1A and Fig. S1) immunofluorescence and flow cytometric characterization of trypsin-isolated cells revealed that these cells maintained the same constitutive markers expression and as evidenced by their wide expression of the tight junction proteins β-catenin, Z0-1 and E-cadherin and the cell adhesion molecules CD54 and CD29, the majority of these cells are from an epithelium-mesothelium origin (Fig. 1B,C and Fig. S2). In further support of these results, the trypsin isolated UtMCs lacked significant expression of endothelial (CD31 and CD106) and haematopoietic (CD45, CD117 and CD11b) cells markers (Fig. 1C and Fig. S2). They also lacked expression of Sca1, a marker strongly expressed by microvascular endothelial cells of the mouse uterine cords (data not shown). Accordingly, trypsinized uterine cords were further confirmed to retain highly intact Sca1^+^ microvascular capillaries (data not shown), suggesting hence that submesothelial vascular structures were not affected during trypsinization. In addition, freshly isolated UtMCs lacked detectable expression of CD44, the receptor for hyaluronic acid, which is constitutively expressed on SMCs [36]. Furthermore, trypsin isolated UtMCs were confirmed to be immunonegative for the SMCs markers α-SMA, calponin, SM22α, caldesmon, SM-myosin and desmin (Fig. S3).

### UtMCs Cultured in SMDM Initiate an EMT and Gain Stemness Characteristics

**Figure 2:**
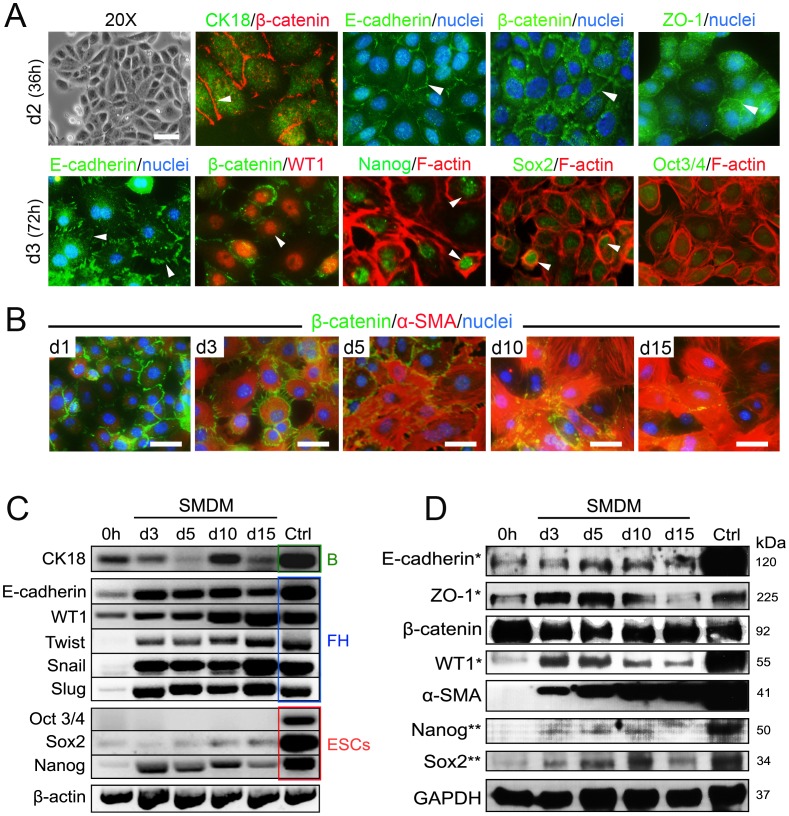
UtMCs cultured in SMDM undergo EMT and express stem and progenitors markers. (**A**) Top panel, UtMCs cultured for 36 hours in SMDM exhibit cobblestone morphology (phase contrast) and express CK18. β-catenin, E-cadherin and ZO-1 were mostly expressed at intercellular contacts (arrowheads). Bottom panel, UtMCs cultured for 72 h in SMDM display irregular intercellular expression of E-cadherin (arrowheads) and strong nuclear expression of WT1 (arrowhead). Majority of UtMCs expressed displayed nuclei positive for Nanog (arrowheads). Sox2 was expressed in less extent in part of the UtMCs examined (arrowheads). UtMCs lacked nuclear immunoreactivity against Oct3/4. (**B**) Time-course immunofluorescence expression of β-catenin (green staining) and α-SMA (red staining) in UtMCs cultured in SMDM for the indicated time periods. Scale bar is 25 µm. (**C**) RT-PCR analysis of mesothelial (CK18, WT1, E-cadherin), EMT (Twist, Snail, Slug) and pluripotent (Oct 3/4, Sox2, Nanog) markers expression in freshly isolated UtMCs (0 h) and UtMCs cultured in SMDM for the indicated time periods. B, are RNAs isolated from adult mouse bladders. FH, are RNAs isolated from E17 mouse fetal hearts. ESCs, are RNAs isolated from mouse D3 ESCs. (**D**) Western blot analysis of freshly isolated UtMCs (0 h) and SMDM cultured UtMCs. For markers labelled with *, control (Ctrl) proteins were isolated from E17 mouse fetal visceral organs (heart, lung, and peritoneal organs). For markers labelled with **, Ctrl proteins were extracted from D3 mouse ESCs. For β-catenin and α-SMA, Ctrl proteins were extracted from adult mouse bladder. (**A–B**) Nuclei are counterstained with Hoechst 33342.

UtMCs displayed cobblestone morphology during their initial 36–48 hours of culture in SMDM. Accordingly, they immunoexpressed CK18 and CK19 (data not shown) and displayed high intercellular expression of β-catenin, ZO-1 and E-cadherin (Fig. 2A and Table 1). In contrast, many of the UtMCs cultured for 72 hours in SMDM lost tight cell-cell contacts and displayed transitional epitheloid-fibroblastoid morphologies (Fig. 2A). Interestingly, at this step UtMCs strongly expressed the transcription factor WT1 and acquired increased F-actin^+^ stress fibers (Fig. 2A). Furthermore, most UtMCs examined partially loss initial tight cell-cell contacts and displayed irregular expression of E-cadherin, β-catenin and ZO-1 (Fig. 2A and Fig. S4). Consistent with their initiation of EMT, the SMDM cultured UtMCs gained expression of α-SMA (Fig. 2B) and expression of the master EMT genes Twist, Snail and Slug (Fig. 2C). Other subset of the SMDM cultured UtMCs was in contrast actively proliferating and displayed Ki-67 positive nuclei (data not shown). Relevantly, UtMCs cultured for 72 hours in SMDM acquired expression of the pluripotent transcription factors Nanog and Sox2 as indicated by RT-PCR, immunofluorescence, and western blot approaches (Fig. 2A,C,D). However, the freshly isolated UtMCs did not express Oct-3/4 as ESCs did.

**Table 1:** Summary of immunofluorescence.

Antigen	Distribution	UtMCs	EMT	UtMCs-derivedVSM-like cells
β-catenin	Epithelial, mesothelial and endothelial cells	**+++**	**+++**	**++**
ZO-1	Epithelial, mesothelial and endothelial cells	**++**	**+++**	+*
E-cadherin	Epithelial and mesothelial cells	**+**	**++**	+*
CK18	Epithelial and mesothelial cells	**+**	**++**	**−**
CK19	Epithelial and mesothelial cells	**+**	**+++**	**−**
WT1	Embryonic mesothelial cells	+*	**+++**	**+**
Nestin	Neural and progenitor cells	**−**	**++**	+*
β-III tubulin	Neurons	**−**	**−**	**−**
Vimentin	Mesenchymal cells	**++**	**+++**	**+++**
Desmin	SMCs, myofibroblasts, cardiomyocytes	**−**	**++**	**++**
α-SMA	SMCs, myofibroblasts, myoepithelial cells	**−**	**++**	**+++**
Calponin	SMCs, myofibroblasts, myoepithelial cells	**−**	**+**	**++**
SM22α	SMCs, myofibroblasts, myoepithelial cells	**−**	**+**	**+++**
SM-myosin	SMCs, myofibroblasts, myoepithelial cells	**−**	**+**	**++**
Caldesmon	SMCs, myoepithelial cells	**−**	**+**	**++**
Smoothelin-B	VSMCs	+*	**++**	**+++**
PDGFR-β	SMCs, myofibroblasts	+*	**++**	**+**
mAChR M3	SMCs, myofibroblasts	**−**	**+**	**++**
mAChR M2	SMCs, myofibroblasts, cardiomyocytes	**+**	**++**	**++**
Gata-4	SMCs, cardiomyocytes	**−**	**++**	**++**
Isl1	Cardiac and SMCs progenitors	**−**	**++**	+*
cTnT	Cardiomyocytes	**−**	**+**	**++**
sACTN	Cardiomyocytes	**−**	**+**	**++**
ANF	Cardiomyocytes	**−**	**−**	+*
CD68	Macrophages, mesothelial cells	+*	**++**	**+**
F4/80	Macrophages	**−**	**−**	**−**
Oct 3/4	ESCs	**−**	**−**	**−**
Nanog	ESCs, MSCs	**−**	**+**	+*
Sox2	ESCs, neural progenitors	**−**	**+**	+*

UtMCs (freshly isolated UtMCs); EMT (UtMCs cultured in SMDM for 5 days); UtMCs-derived VSM-like cells (UtMCs cultured for 15 days in SMDM). Antigen expression was evaluated as being negative (–), very weak (+*), weak (+), intermediate (++) or strong (+++).

Alpha smooth muscle actin (α-SMA); Atrial natriuretic factor (ANF); Cytokeratin 18 CK18); Cytokeratin 19 (CK19); Cardiac troponin T (cTnT); Embryonic stem cells (ESCs); Muscarinic acetylcholine receptors (mAChR); Mesenchymal stem cells (MSCs); Platelet derived growth factor receptor beta (PDGFR-β); Sarcomeric alpha actinin (sACTN); Wilms tumor protein 1 (WT1); Zona Occludens-1 (ZO-1).

### UtMCs Cultured in SMDM Acquire Vascular Smooth Muscle Markers Expression

**Figure 3:**
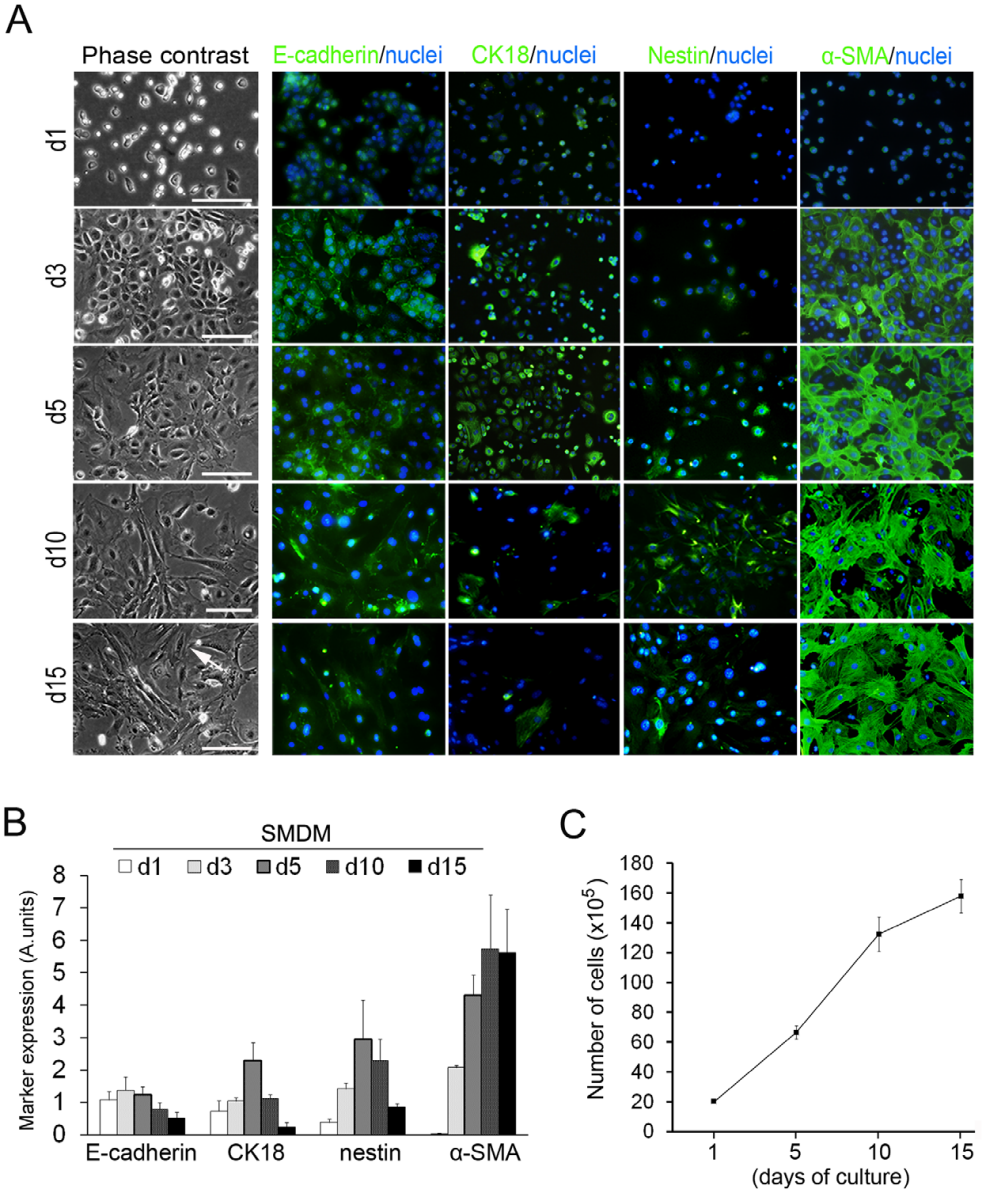
Immunofluorescence analysis of the vasculogenic differentiation of UtMCs cultured in SMDM. (**A**) Left images show phase-contrast pictures of UtMCs cultured in SMDM for the indicated time periods. Scale bar is 50 µm. Right images show corresponding immunofluorescence expression of E-cadherin, CK18, α-SMA and nestin. Nuclei are counterstained in blue with Hoechst 33342. (**B**) Summary of MetaMorph-based fluorescence signals quantification of E-cadherin, CK18, nestin and α-SMA. Data are shown as marker fluorescence ± s.d., as deduced from immunofluorescent images performed onto 3 independent cultures. (**C**) Expansion of UtMCs during 15 days of culture in SMDM as deduced after calculation of cumulating numbers of cells generated after 10 and 15 days of culture.

The detection of low to intermediate immunoreaction of α-SMA, desmin, SM-myosin and caldesmon in UtMCs cultured for 36 hours in SMDM suggested that their vasculogenic differentiation was rapidly induced in SMDM culture (Fig. S3). At this step, calponin and SM22α were however not detected. Immunofluorescence analysis of the SMDM cultured UtMCs (Fig. 3A,B) indicated that UtMCs initially differentiated into [E-cadherin^+^/CK18^+^/nestin^+^/α-SMA^+^] cells. This transient EMT immunophenotype was however lost in subculture, when UtMCs massively adopted the morphology and immunophenotype [E-cadherin^−/^CK18^−/^nestin^−/^α-SMA^+^] of SM-like cells. Double immunofluorescence labellings of UtMCs undergoing EMT confirmed their high coexpression of epithelial (CK18 and CK19) and SM (α-SMA, SM22α, SM-myosin and desmin) markers (Fig. S5).

**Figure 4:**
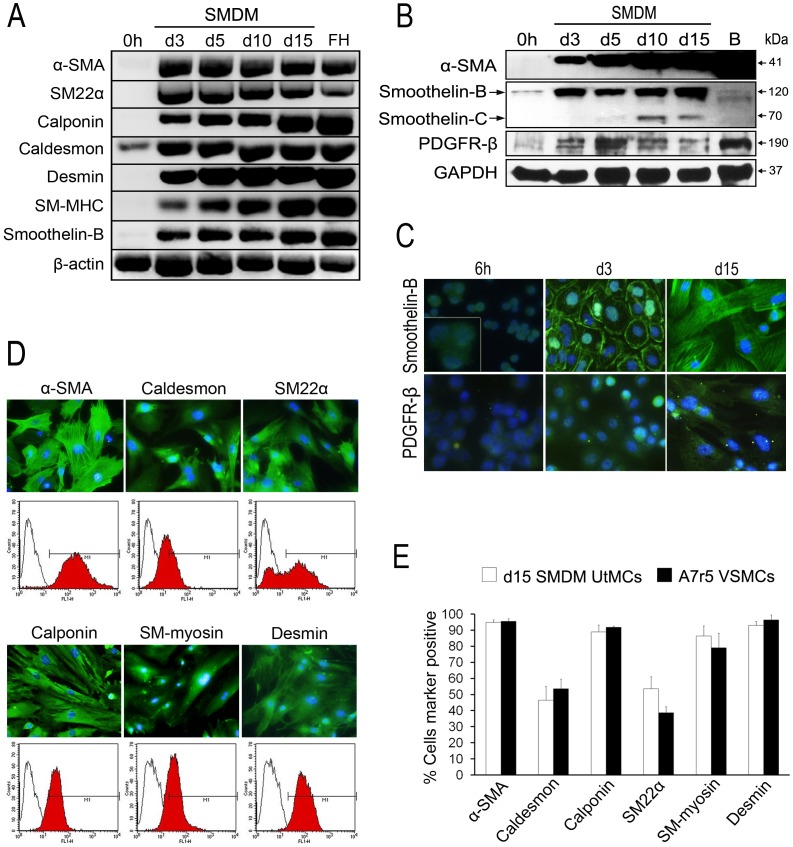
UtMCs cultured in SMDM acquire VSMCs markers expression. (**A**) RT-PCR analysis of SM genes expression in freshly isolated UtMCs (0 h) and UtMCs cultured in SMDM for the indicated time periods. FH, are RNAs isolated from E17 mouse fetal hearts. (**B**) Western blot analysis of α-SMA, smoothelin-B and PDGFR-β expression in freshly isolated UtMCs (0 h) and UtMCs cultured in SMDM for the indicated time periods. B, are proteins extracted from adult mouse bladders. Note that smoothelin-B antibody (H-300) also detected an intermediate smtn-C isoform in SMDM cultured. (**C**) Immunofluorescence detection of smoothelin-B and PDGFR-β in UtMCs cultured for the indicated time periods in SMDM. (**D**) Immunofluorescence and intracellular flow cytometric expression of SM markers in UtMCs cultured for 15 days in SMDM (d15 SMDM cultured UtMCs). Red histograms are cells incubated with primary and secondary antibodies. Black line histograms are cells incubated with secondary antibody alone. (**E**) Summary results of intracellular flow cytometric quantification of SM markers positive cells in d15 SMDM cultured UtMCs and A7r5 VSMCs. Results are represented as mean percentages of cells marker positive ± s.d. from 3 independent cultures. (**C**, **D**), nuclei are counterstained in blue with Hoechst 33342.

RT-PCR analysis of SM genes expression in the freshly isolated UtMCs confirmed their lack of detectable mRNAs encoding for α-SMA, calponin, SM22α, desmin, SM-MHC and smoothelin-B (Fig. 4A). They were however found to express mRNAs encoding for h-caldesmon, a protein that is constitutively expressed in epitheloid mesotheliomas [37]. Consistent with the initiation of their vasculogenic differentiation in SMDM culture, UtMCs cultured for 3 days in SMDM exhibited a robust expression of α-SMA, calponin, SM22α, desmin SM-MHC and smoothelin-B (Fig. 4A).

Western Blotting analysis indicated that α-SMA was not expressed in the freshly isolated UtMCs but was however robustly induced in time-dependent manner (Fig. 4B). Unexpectedly, the freshly isolated UtMCs were however found to weakly express smoothelin-B as shown by western blot and immunofluorescence experiments (Fig. 4B,C). Smoothelin-B expression was detected in small cytoplasmic granules in the vast majority of cells examined (Fig. 4C). However, and consistent with their initiation of vasculogenic differentiation in SMDM culture, smoothelin-B expression was found to increase in the UtMCs cultured for 3 days in SMDM. Furthermore, in subculture, UtMCs-derived VSM-like cells strongly expressed smoothelin-B in the typical stress fiber pattern of SMCs [34], [35]. Interestingly, the SMDM cultured UtMCs also gained expression of a smaller smoothelin isoform with a molecular weight of (±70 kDa) that might correspond to the smtn-C isoform, which is transiently expressed in the developing cardiac and skeletal muscle during chicken embryogenesis [38]. Finally, and consistent with their vasculogenic differentiation, both western blotting and immunofluorescence analysis indicated that the SMDM cultured UtMCs express PDGFR-β (Fig. 4B,C).

Next, we used immunofluorescence and flow cytometry to determine the SM expression pattern of UtMCs cultured 15 days in SMDM (Fig. 4D,E). Closely resembling to A7r5 VSMCs, 90% of the SMDM cultured UtMCs were found to express α-SMA, calponin, SM-myosin and desmin. In lesser extent, 50% of caldesmon and SM22α expression was detected in both UtMCs and A7r5 VSMCs.

Analysis of the surface marker profile of the SMDM cultured UtMCs was also performed (Fig. S2). Consistent with the induction of their EMT, CD54 expression was found to be significantly reduced (*P<0.01*) on the UtMCs-derived VSM-like cells when compared to trypsin isolated UtMCs (*P<0.01*). In contrast, UtMCs-derived VSM-like cells exhibited significant increase of CD44 expression (*P<0.01*), a marker constitutively expressed on SMCs [36].

### UtMCs Cultured in SMDM Express Cardiac Muscle Markers

**Figure 5:**
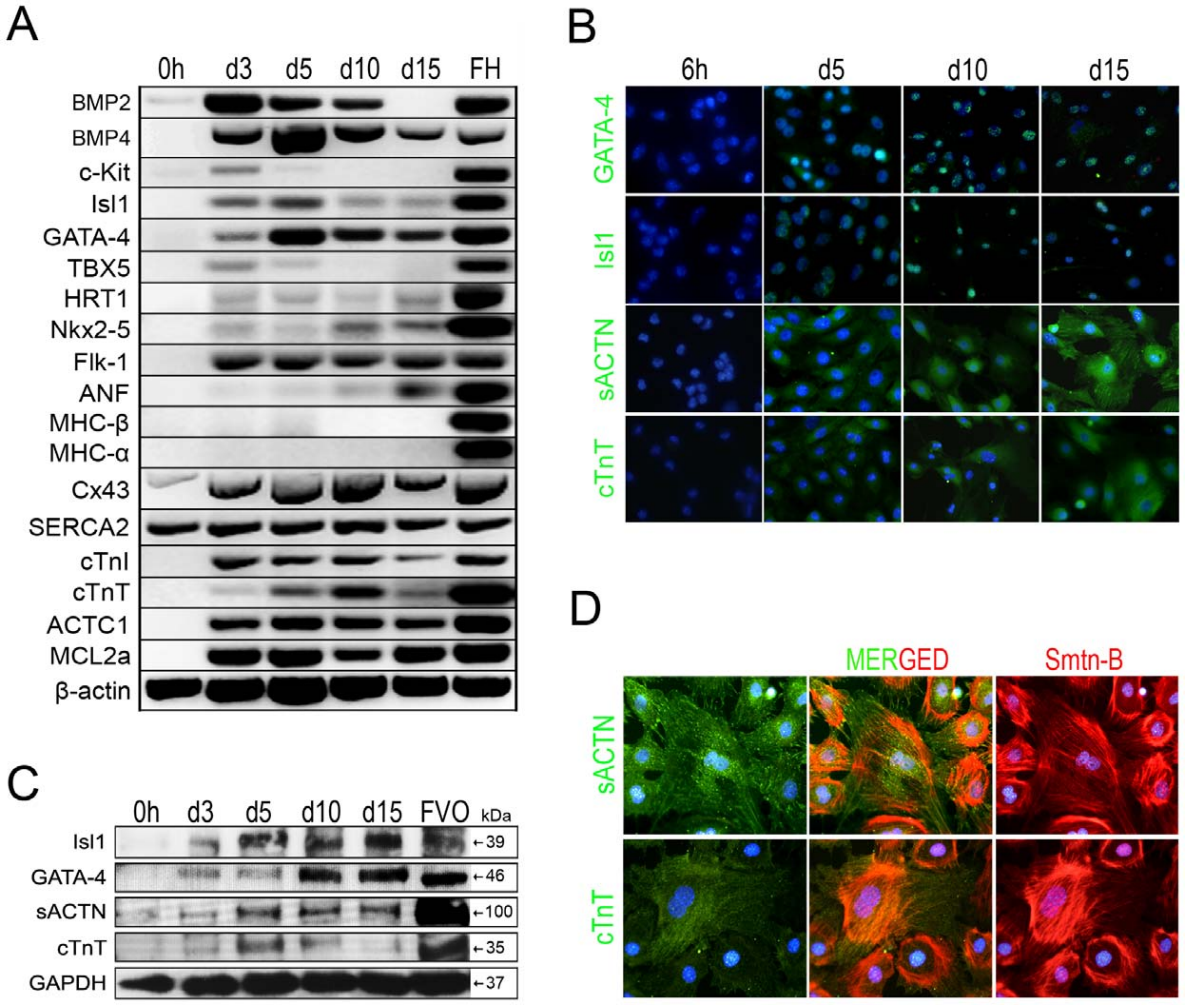
UtMCs cultured in SMDM express cardiac lineage markers. (**A**) RT-PCR analysis of cardiac genes expression in freshly isolated UtMCs (0 h) and UtMCs cultured for 3, 5, 10 and 15 days in SMDM. FH, are RNAs isolated from E17 mouse fetal hearts. (**B**) Immunofluorescence expression of Gata-4, Isl1, sACTN and cTnT in UtMCs cultured for 6 h and 5, 10 and 15 days in SMDM (**C**) Western blot analysis of Isl-1, GATA-4, sACTN and cTnT expression in freshly isolated UtMCs (0 h) and UtMCs cultured for 3, 5, 10 and 15 days in SMDM. FVO, are proteins extracted from E17 mouse fetal visceral organs (heart, lung, and peritoneal organs). (**D**) Double immunofluorescence detection of smoothelin-B with either sACTN or cTnT in UtMCs cultured 10 days in SMDM. (**B**, **D**) Nuclei are counterstained in blue with Hoechst 33342.

Gene analysis of the SMDM differentiating UtMCs cultures also revealed their gain of expression of the transcription factors Isl1 and Gata-4 (Fig. 5A), which are both robustly expressed in multipotent cardiac progenitors [39]–[43]. In further support of their gain of cardiomyogenic traits, the SMDM cultured UtMCs also expressed BMP2 and BMP4, two morphogens that are critically required for the cardiac proepicardial development [44], [45] and myocardial differentiation [46]. In addition, the SMDM cultured UtMCs also expressed Flk-1, the vascular endothelial growth factor receptor type 2 (VEGFR2) that is strongly expressed in multipotent cardiovascular progenitor cardiomyocytes [41] and gained detectable expression of ACTC1, cTnI, cTnT, MLC2a and ANF, which are all components of the cardiomyocytic contractile machinery. Connexin 43, the main gap junction protein in the myocardium, was also strongly upregulated. Contrary to a real cardiomyogenic differentiation, the cardiac progenitor markers c-Kit and Tbx5 were minimally detected in the UtMCs that were cultured for 3 and 5 days in SMDM and not onward. Furthermore, the SMDM cultured UtMCs weakly expressed HRT1 and Nkx2.5 and lacked detectable expression of MHC-α and MHC-β.

The expression pattern of Gata-4, Isl1, sACTN and cTnT was also determined by western blot and immunofluorescence (Fig. 5B,C). Gata-4, Isl1, sACTN and cTnT were expressed after 3 days of culture in SMDM and their expression was up-regulated at 10 days. Interestingly, the expression of cTnT increased at day 5 and then decreased in UtMCs subcultured in SMDM. Unexpectedly, almost all SMDM cultured UtMCs examined co-immunoexpressed the VSMCs marker smoothelin-B and the cardiac markers sACTN and cTnT, this finding could unequivocally demonstrate that these cells had acquired dual leiomyogenic and cardiomyogenic characteristics (Fig. 5D).

### UtMCs-derived VSM-like cells Acquire Functional mAChRs

SMCs express functional mAChRs, being of predominant expression those of the M2 subtype, even if excitation-contraction coupling is principally mediated through M3 mAChRs in most SMCs [47].

**Figure 6:**
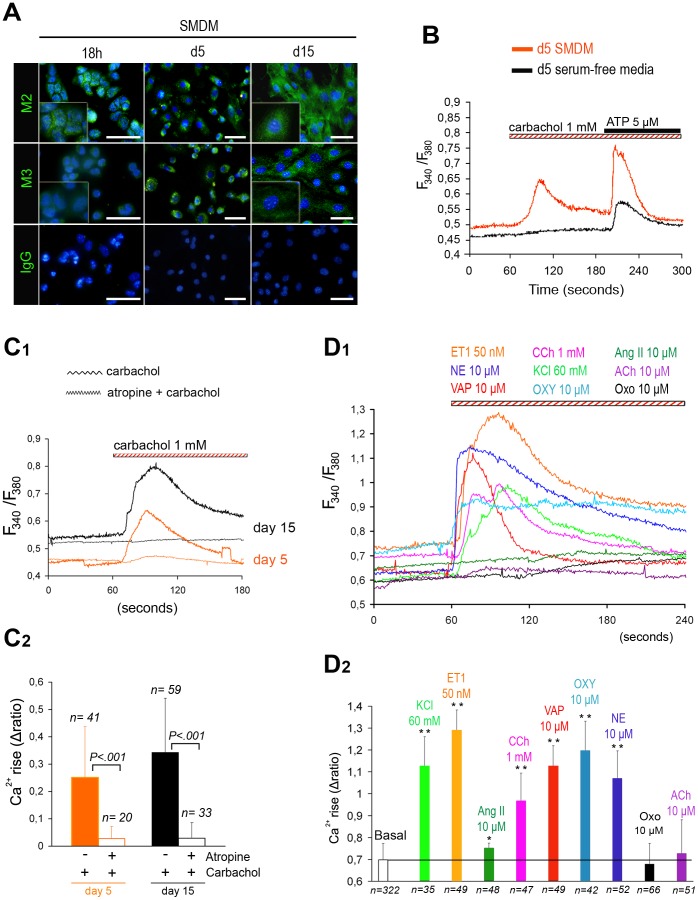
UtMCs-derived VSM-like cells display [Ca^2+^]*_i_* **rises against several vasoactive agonists.** (**A–C**) SMDM cultured UtMCs acquire functional muscarinic receptors (mAChRs). (**A**) Representative immunoexpression of M2 and M3 mAChRs in UtMCs cultured in SMDM for 18 h, 5 days (undergoing EMT) and 15 days (UtMCs-derived VSM-like cells). Nuclei are counterstained in blue with Hoechst 33342. Scale bar is 50 µm. (**B**) Representative traces of cytosolic Ca^2+^ concentrations (represented as fura-2 ratio, F_340_/F_380_) recorded in UtMCs cultured for 5 days in SMDM (induced EMT) or in serum-free media (mesothelial phenotype) after their sequential exposure to 1mM carbachol and 5 µM adenosine triphosphate (ATP). (**C1–C2**) UtMCs cultured in SMDM exhibit an atropine-sensitive Ca^2+^ response to carbachol (**C1)** Representative traces of [Ca^2+^]*_i_* recorded in UtMCs cultured for 5 and 15 days in SMDM that were preincubated or not with 1 mM atropine (1 mM) and then challenged against 1 mM carbachol. (**C2)** Summary data of [Ca^2+^]*_i_* rises (Δratio ± s.d.) calculated from indicated number (n) of cells tested. (**D1–D2**) UtMCs-derived VSM-like cells exhibit [Ca^2+^]*_i_* rises to other several vasoactive agonists. (**D1)** Representative traces of [Ca^2+^]*_i_* recorded in UtMCs-derived VSM-like cells that were incubated against 60 mM high K^+^ (KCl), 50 nM endothelin 1 (ET1), 10 µM angiotensin II (Ang II), 1 mM carbachol (CCh), 10 µM vasopressin (VAP), 10 µM oxytocin (OXY),10 µM noradrenaline (NE), 10 µM Oxotremorine (OXO) and 10 µM acetylcholine (ACh). (**D2)** Summary results of vasoactive agonists-induced [Ca^2+^]*_i_* rises (Δratio ± s.d.) calculated from indicated number (n) of cells tested (*, indicates *p*<0.05 and **p<0.001 by comparison to basal Δratio).

Immunofluorescence analysis of the freshly isolated UtMCs indicated that they expressed weak amounts of M2 mAChRs, whereas M3 mAChRs were difficulty detected above background levels (Fig. 6A). UtMCs that were cultured for 5 days in SMDM were in contrast found to immunoexpress M3 mAChRs and displayed significant rises in cytosolic calcium [Ca^2+^]*_i_* in response to 1 mM of the nicotinic and mAChRs agonist carbachol (Fig. 6B). UtMCs that were maintained for 5 days in a serum-free media retained original mesothelial morphologies (data not shown) and lacked detectable carbachol-induced [Ca^2+^]*_i_* rises (Fig. 6B).

Accordantly to their vasculogenic differentiation, the UtMCs cultured for 15 days in SMDM were found to display stronger carbachol-induced [Ca^2+^]*_i_* rises (0.878±0.225) than those cultured for only 5 days (0.704±0.163, *P<0.03*) in SMDM (Fig. 6C). Finally, the UtMCs that were preincubated with 1 mM atropine (selective mAChRs antagonist) lacked [Ca^2+^]*_i_* rises against carbachol, confirming that carbachol was mediating its effect through mAChRs and not nicotinic receptors (Fig. 6C).

### UtMCs-derived VSM-like Cells Display Vasoactive Agonists-induced [Ca^2+^]*_i_* Oscillations

Next, we determined whether UtMCs-derived VSM-like cells might display [Ca^2+^]*_i_* rises to other vasoactive agonists (Fig. 6D1,2). We found that UtMCs-derived VSM-like cells could display from intermediate to potent [Ca^2+^]*_i_* rises in response to 50 nM endothelin 1, 10 µM oxytocin, 60 mM high K^+^, 10 µM arginin-vasopressin, 10 µM norepinephrine, indicating they have acquired VSMCs properties. Weak [Ca^2+^]*_i_* rises were also detected in response to 10 µM angiotensin II (10 µM). Finally, UtMCs-derived VSM-like cells lacked detectable [Ca^2+^]*_i_* rises when exposed to 10 µM of the cholinergic agonists acetylcholine and oxotremorine.

### UtMCs-derived VSM-like Cells Display Vasoactive Agonists-induced Contraction

**Figure 7:**
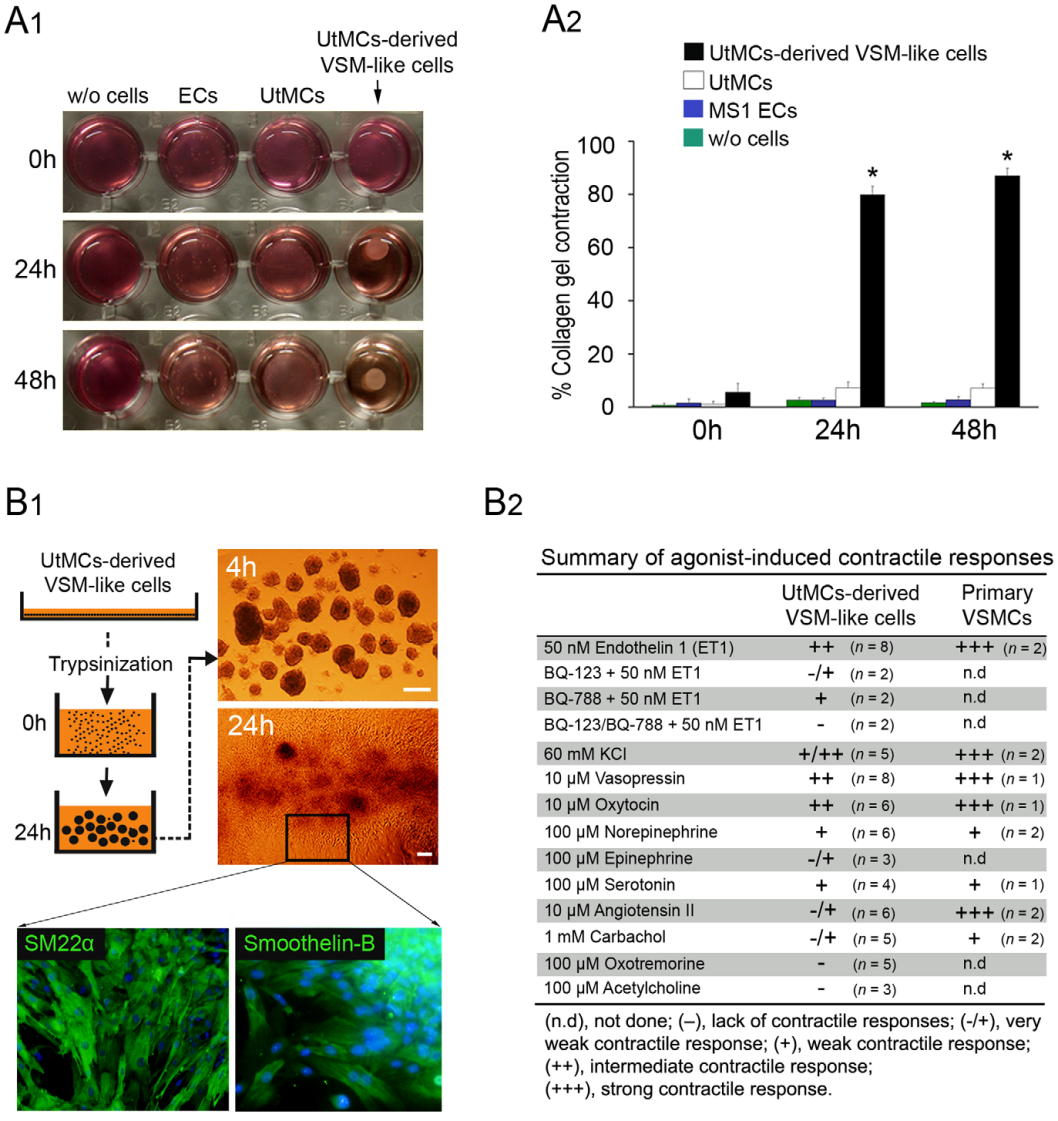
UtMCs-derived VSM-like cells display a contractile phenotype. (**A1–A2**) Cells-collagen gel lattices contraction assay. (**A1**) Shows picture of collagen gel lattices lacking cells (w/o cells) or seeded with MS1 endothelial cells, freshly isolated UtMCs (UtMCs) and UtMCs-derived VSM-like cells after 0, 24 and 48 hours (h) of their initial release from the well. (**A2**) Summary of the collagen gel lattices contraction assay. Data indicate the mean percentile reduction in lattices area ± s.d. from 3 independent experiments (*, *p*<0.001 compared to initial area). (**B1–B2**) UtMCs-derived VSM-like cells display vasoactive agonists-induced contractile responses. (**B1**) Schematic protocol used to generate UtMCs-derived VSM-like cells spheroids. Right images, spheroids adhered for 4 hours (spreading) and 24 hours (multilayered culture). Multilayered UtMCs-derived VSM-like cells cultures retain SM22α and smoothelin-B immunofluorescence expression. (**B2**) Shows summary results of vasoactive agonists-induced contractile responses displayed by UtMCs-derived VSM-like cells. Contractile responses displayed by primary VSMCs to several vasoactive agonists are shown for comparison. (n), number of independent cultures tested.

Primary SMCs and SMCs derived from ESCs [22] and from mesenchymal stem cells (MSCs) [48] could generate sufficient mechanical force to compact collagen lattices in a time-dependent manner. As described by Kim et al. [48] we used a similar cells-collagen lattices assay to test whether the SMDM cultured UtMCs could acquire similar contractile properties. As expected, the freshly isolated UtMCs failed to contract the collagen lattices. In contrast and similar to other SMCs populations, the UtMCs-derived VSM-like cells-collagen lattices could contract 80% of their original area within 24 hours (Fig. 7A1,2).

The first function of VSMCs is to contract and relax in response to vasoconstrictors and vasodilators, respectively. To test whether the UtMCs-derived VSM-like cells had acquired functional properties of VSMCs we sought to analyse their capacity to contract in response to several vasoactive agonists (Fig. 7B1,2). To better mimic native SM tissues organization, we generated multilayered UtMCs-derived VSM-like cells cultures (Fig. 7B1) and used time lapse image recording to monitor their contraction. Contraction intensities of the UtMCs-derived VSM-like cells were compared to the strong contraction of primary VSMCs when induced with ET1, KCl, vasopressin, oxytocin and angiotensin II (see Fig. 7B2 for summary of contractile responses). Remarkably, UtMCs-derived VSM-like cells reproducibly displayed intermediate contraction when incubated with 50 nM endothelin-1 (Movie S1), 10 µM vasopressin (Movie S2) and 10 µM oxytocin (Movie S3) and intermediate to weak contraction when treated with 60 mM high K^+^ (Movie S4), 10 µM norepinephrine (movie not shown), 10 µM serotonin (movie not shown) and 10 µM epinephrine (movie not shown). In contrast, only very weak contractile responses were registered against 1 mM carbachol (movie not shown) and 10 µM Angiotensin II (Movie S5). Finally, the UtMCs-derived VSM-like cells lacked contractile activity against 100 µM oxotremorine (movie not shown) and 100 µM acetylcholine (movie not shown). In this study we did not test vasodilators drugs.

### Endothelin-induced Contraction of UtMCs-derived VSM-like Cells is Principally Mediated via ET_A_ Receptors

In mammals, two subtypes of endothelin receptors (ET_A_ and ET_B_) have been cloned. Studies using the selective ET_A_ and ET_B_ receptors antagonists indicated that the vasoconstrictor activity of endothelin-1 (ET1) is primarily mediated through ET_A_ receptors and in much lower way via ET_B_ receptors in most SMCs [49]. Therefore, we sought to evaluate whether UtMCs-derived VSM-like cells had acquired similar contractile mechanism. Summary analysis of contractile movies (Fig. 7B2) indicated that UtMCs-derived VSM-like cells preincubated simultaneously with 1 µM BQ-123 (ET_A_ antagonist) and 1 µM BQ-788 (ET_B_ antagonist) lacked detectable contractile responses against 50 nM ET1 (Movie S6). In contrast, UtMCs-derived VSM-like cells that were preincubated with 1 µM BQ-788 alone displayed intermediate to strong contractile responses against 50 nM ET1 (Movie S7). Supporting the concept that the ET1-induced contraction of UtMCs-derived VSM-like cells may be mediated principally via ET_A_ receptors and in lower extent via ET_B_ receptors, UtMCs-derived VSM-like cells preincubated with 1 µM BQ-123 alone could only display very weak contraction against 50 nM ET1 (Movie S8).

## Discussion

This study reports for the first time the isolation and characterization of primary uterine MCs (UtMCs). This study also shows for the first time that UtMCs retain the capacity to acquire molecular and functional properties of differentiated contractile VSMCs. To the best of our knowledge, none of the adult MCs analyzed so far were reported to be capable to gain functional characteristics of VSMCs. This goal was achieved by culturing UtMCs in a high glucose based media containing 10% fetal bovine serum, 20 ng/ml EGF and 1 ng/ml hydrocortisone. Ahmed et al. previously suggested that hydrocortisone could potentiate in vitro the EGF-induced EMT of the human ovarian surface epithelium which is a mesothelium in nature and anatomically very closer to the uterine mesothelium, moreover the authors reported that this induction was associated with the activation of both the Erk and ILK pathways [50]. The distinction between established pathways implicated in EMT remain difficult, as there may be significant cross-talk between these pathways that offer a cooperative scenario for induction and specification of EMT within a particular tissue, in our case, both ILK/Erk1/2, PI3/Akt and Smad-dependent (Smad2/3) pathways are activated (data not shown). Despite these interesting findings, we did not addressed whether the transdifferentiated ovarian MCs might have acquired molecular and functional characteristics of cells of the vascular SM lineage.

As additional relevant novel finding, we show for the first time that UtMCs that were undergoing EMT and proliferation gained expression of the stem and progenitor cells markers Nanog, Sox2, Isl1 and nestin. Interestingly, Nanog and Sox2 are two master genes controlling the self-renewal and pluripotency of ESCs. In addition, they are also expressed in multipotent adult MSCs and mesodermal progenitor cells [51], [52] and epithelial cells undergoing EMT [53], [54]. Here, Nanog, Sox2 and WT1 were maximally expressed during early and middle EMT steps of the SMDM cultured UtMCs, which were the steps corresponding to their early vasculogenic differentiation and proliferation. The cardiovascular progenitor cells markers Gata-4 and Isl1 were in contrast differentially expressed at middle and late EMT steps, when UtMCs had massively transdifferentiated towards VSM-like cells.

Interestingly, a previous work indicated that WT1 and Gata-4 are coexpressed in the E12.5 mouse embryonic liver mesothelium, at step of liver embryogenesis, when WT1^+^/Gata-4^+^ MCs delaminate from the liver surface and migrate in stromal layers where they differentiate into stellate cell progenitors and VSMCs [55]. Wt1-Cre genetic mesothelial lineage marking in the developing mouse heart, lung, liver and gut provided definitive proof that WT1-expressing MCs undergo EMT and give rise to αSMA+ VSMCs [6], [8]–[10]. Indeed, Wt1-null mouse mutant embryos exhibited defective heart development due to an impaired mesothelial EMT and production of vascular progenitors [56].

The finding of Isl1 expression in the SMDM cultured UtMCs is very interesting because it marks a population of multipotent cardiovascular progenitors in the developing heart [40] and ESCs differentiating cultures [41] that give rises to cardiomyocytes, VSMCs and vascular endothelial cells. The finding that Isl1 is still expressed in αSMA^+^ VSMCs progenitors of the proximal aorta and pulmonary artery trunk of mouse E12.5 embryonic hearts [42] could therefore support the idea that the Isl1^+^/αSMA^+^ differentiating UtMCs had acquired characteristics of early VSMCs progenitors. Furthermore, most of the αSMA^+^ SMDM differentiating UtMCs were also found to express nestin, a marker for neural progenitors that is transiently expressed in VSMCs progenitors of rat embryonic arteries and lost in differentiated VSMCs [57].

As other key finding in this study, we report that the SMDM cultured UtMCs robustly gained expression of PDGFR-β. Interestingly, PDGFR-β signalling through its ligand PDGF-BB induces both mitogenic [58] and potent EMT-inducing activity [15], [19] onto cultured adult MCs. PDGF signalling is also critical for the epicardial function and neovasculogenesis in the regenerating zebrafish heart [59]. In addition, mouse embryos mutants carrying a specific deletion of PDGFR-β in their epicardium were found to develop severe defective coronary vessels and to lack coronary VSMCs due to an impaired migration of proepicardial-derived cells [60], [61].

Relevantly, we show that the SMDM cultured UtMCs could acquire expression of the SMCs markers αSMA, SM22α, SM-myosin, caldesmon, calponin, desmin and smoothelin-B. Interestingly, the smoothelin-B isoform is thought to be only expressed in contractile SMCs of vascular tissues in adults [34]. The critical role of smoothelin-B in maintaining the contractile phenotype of VSMCs was evidenced as smoothelin-B^−/−^ mutant mice displayed reduced arterial contractility [35]. Interestingly, the SMDM cultured UtMCs also expressed a smaller smoothelin isoform (±70 kDa) which molecular weight could correspond to the previously reported smoothelin-C isoform that was found to be transiently expressed during embryonic cardiac and skeletal muscle development [38].

Supporting the possibility that the SMDM cultured UtMCs had also gain cardiomyogenic traits was further demonstrated by RT-PCR detection of their expression of the cardiac progenitor markers HRT1, C-kit, Tbx5, Gata-4 and Nkx2.5 and markers of early cardiac development (BMP2, BMP4). In addition, their expression of other cardiac markers (Cx43, ANF and SERCA2) and cardiac contractile proteins ACTC1, sACTN, Cx43, cTnI, cTnT and MLC2a could definitively evidence their gain of cardiomyogenic properties. Their lack of expression of some stringent cardiac contractile proteins such as MHC-α and MHC-β may however demonstrate that these cells have only acquired partial features of cardiomyocytes.

Supporting the expression of cardiac markers in the SMDM cultured UtMCs, van Tuyn et al previously indicated that adult human epicardial MCs cultured in serum-containing media could spontaneously undergo EMT and coexpress αSMA, Gata-4 and cTnT [14]. Their adenoviral transduction with myocardin, a master transcription factor of SM differentiation [62] could additionally induce their expression of SM-MHC, SM22α, sACTN, MLC2a, MHC-α and MHC-β. These epicardial-derived SM-like cells however lacked expression of Nkx2.5 and cTnI mRNAs transcripts. It is also noteworthy that the SMDM cultured UtMCs could express SM-MHC, SM22α, sACTN and MLC2a without the need of forced myocardin expression. Collectively, these results might suggest that adult MCs retain an intrinsic bipotency to perform leiomyogenic and cardiomyogenic differentiation.

The capacity of UtMCs-derived VSM-like cells to exhibit potent [Ca^2+^]*_i_* rises and significant contractile responses to voltage [high K^+^] and vasoactive agonists [endothelin 1, vasopressin and oxytocin] might represent the strongest evidence that UtMCs have acquired functional contractile mechanisms that are found in adult mature VSMCs. Interestingly, oxytocin, vasopressin and endothelin-1 are among the most potent vasoconstrictors described so far. Oxytocin was initially described as a potent constrictor for uterine myometrial SMCs and as such is primarily used in partirution. However, oxytocin and its analogue vasopressin can also provoke a potent vasoconstriction of uterine arteries of different species, by acting via their own receptors, even if a partial cross-activation could be detected, because oxytocin can bind to the vasopressin receptor V1A [63], [64]. In addition, oxytocin receptors were also detected in cava and pulmonary veins, pulmonary artery and aorta suggesting that the vasculature contains an intrinsic oxytocin system [65]. We also found that the contractile activity of endothelin-1 (ET-1) in the UtMCs-derived VSM-like cells is mediated principally via ET_A_ receptors and in lower extent via ET_B_ receptors. Interestingly, even though ET-1-induced vasoconstriction is primary mediated through ET_A_ receptors in most SMCs [49] only a combined blockade of both ET_A_ and ET_B_ receptors can abolish their ET-1-induced contraction [66].

The fact that UtMCs-derived VSM-like cells also displayed intermediate to weak functional responses against norepinephrine, epinephrine, serotonin, carbachol and angiotensin II indicate that they have acquired a wide range of contractile mechanisms of the differentiated contractile VSMCs. It was however noteworthy that the UtMCs-derived VSM-like cells may have acquired only limited numbers of functional mAChRs as they required high concentrations of carbachol (1 mM) to display significant [Ca^2+^]*_i_* rises.

## Supporting Information

Figure S1 **Whole mount-immunofluorescence characterization of the uterine mesothelium.** (**A**) Photographs showing mouse uterine cords before and after mechanical separation from uterine fat pads. (**B**) Shows whole-mount immunofluorescence characterization of the mouse uterine mesothelium. Outermost uterine mesothelium layer exhibits expression of tight junction proteins (β-catenin, ZO-1 and E-cadherin) and of stromal/mesenchymal markers (CD29, CD54 and vimentin). In contrast, mesothelium appeared immunonegative against WT1, CD44 and α-SMA. Nuclei are counterstained in blue with Hoechst 33342.
(TIF)

Figure S2 **Surface marker profile analysis the freshly isolated and SMDM cultured UtMCs.** (**A**) Freshly isolated UtMCs and UtMCs cultured in SMSM for 5 days (undergoing EMT) and 15 days (UtMCs-derived VSM-like cells) were characterized by flow cytometry. Panel shows representative histograms obtained for each marker. Pink histograms correspond to cells incubated with fluorescent-conjugated antibodies. Green histograms correspond to cells incubated with fluorescent-conjugated isotype-matched antibodies. (**B**) Summary results of percentages of cells marker positive ± s.d. Significant changes in cell surface marker expression were analyzed after 5 and 15 days of culture in SMDM and compared against values found in freshly isolated UtMCs; *, *p*<.05; **, *p*<.03; ***, *p*<.01.
(TIF)

Figure S3 **UtMCs rapidly acquire SMCs markers expression upon culture in SMDM.** Upper and lower immunofluorescence panels show immunofluorescence expression levels of the SMCs markers; α-SMA, calponin, SM22α, desmin, SM-myosin and caldesmon in UtMCs cultured for 6 and 36 hours in SMDM. Nuclei are counterstained in blue with Hoechst 33342. Scale bar is 50 µm.
(TIF)

Figure S4 **UtMCs cultured for 3 days in SMDM display epithelial type ZO-1 and β-catenin expression pattern.** Immunofluorescence of ZO-1 (upper image) and β-catenin (lower image) in UtMCs cultured for 3 days in SMDM allow the visualization of UtMCs that have detached from each other (arrowheads) and that present a partial or total loss of ZO-1 and β-catenin expression. Areas with higher cell densities seeding (centre of the well) show UtMCs displaying tight cohesion and vivid intercellular immunofluorescence expression of ZO-1 and β-catenin (white dashed areas).
(TIF)

Figure S5 **SMDM differentiating UtMCs coexpress epithelial and SM markers.** (**A**) UtMCs cultured for 5 days in SMDM were double immunofluorescently labelled against the epithelial/mesothelial marker CK18 and the SMCs markers; α-SMA, SM-myosin and desmin. Arrows show cells co-expressing CK18 and SMCs markers. (**B**) UtMCs cultured for 10 days in SMDM were double immunofluorescently labelled against the epithelial/mesothelial marker CK19 and the SMCs markers; α-SMA and SM22α. Arrowheads point to UtMCs retaining a mesothelial phenotype (CK19^+^/α-SMA^-^ and CK19^+^/SM22α^-^). Arrows show UtMCs-derived VSM-like cells that are detected as CK19^+^/α-SMA^+^ and CK19^+^/SM22α^+^ cells. (A–B) Nuclei are counterstained in blue with Hoechst 33342 and scale bar is 50 µm.
(TIF)

Table S1 List of primary antibodies used in this study.
(DOC)

Table S2 List of secondary antibodies used in this study.
(DOC)

Table S3 List of fluorescent conjugated antibodies used in this study.
(DOCX)

Table S4 List of primers used for Reverse Transcriptase-Polymerase Chain Reaction.
(DOCX)

Movie S1 **Movie showing contraction of UtMCs-derived VSM-like cells when incubated with 50 nM endothelin-1.**
(MP4)

Movie S2 **Movie showing contraction of UtMCs-derived VSM-like cells when incubated with 10 µM vasopressin.**
(MP4)

Movie S3 **Movie showing contraction of UtMCs-derived VSM-like cells when incubated with 10 µM oxytocin.**
(MP4)

Movie S4 **Movie showing contraction of UtMCs-derived VSM-like cells when incubated with 60 mM KCl.**
(MP4)

Movie S5 **Movie showing contraction of UtMCs-derived VSM-like cells when incubated with 10 µM Angiotensin II.**
(MP4)

Movie S6 **Movie showing contraction of UtMCs-derived VSM-like cells when preincubated with (1 µM BQ-123 + 1 µM BQ-788) and then incubated with 50 nM endothelin-1.**
(MP4)

Movie S7 **Movie showing contraction of UtMCs-derived VSM-like cells when preincubated (1 µM BQ-788) and then incubated with 50 nM endothelin-1.**
(MP4)

Movie S8 **Movie showing contraction of UtMCs-derived VSM-like cells when preincubated with (1 µM BQ-123) and then incubated with 50 nM endothelin-1.**
(MP4)
