# Supplementary figures and images for: Functional Vascular Smooth Muscle-like Cells Derived from Adult Mouse Uterine Mesothelial Cells
Source: PLoS One. 2013 Feb 6;8(2):e55181. doi: 10.1371/journal.pone.0055181 (PMC3566215; doi:10.1371/journal.pone.0055181)

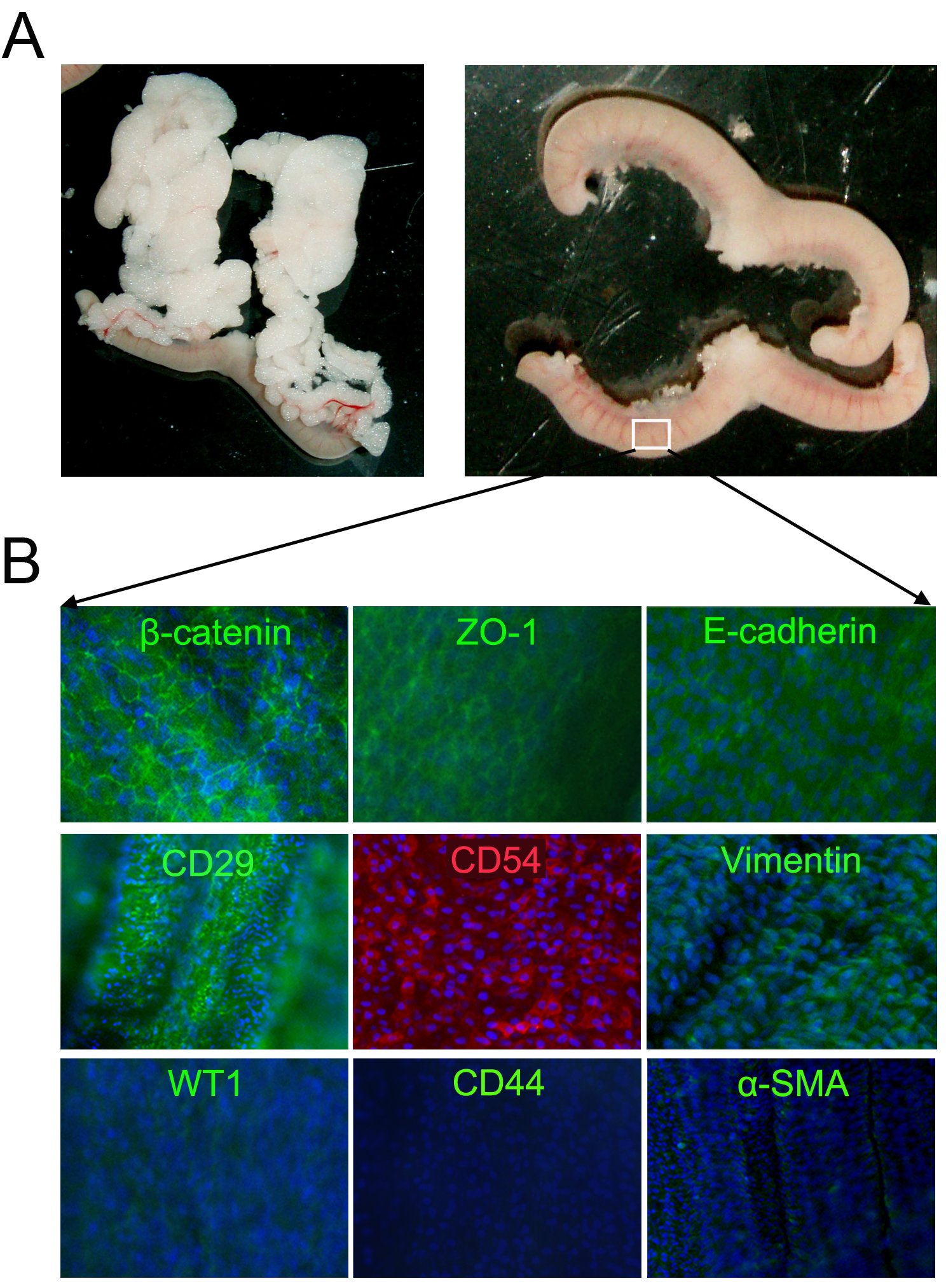

Supplement: Figure S1 — Whole mount-immunofluorescence characterization of the uterine mesothelium. (A) Photographs showing mouse uterine cords before and after mechanical separation from uterine fat pads. (B) Shows whole-mount immunofluorescence characterization of the mouse uterine mesothelium. Outermost uterine mesothelium layer exhibits expression of tight junction proteins (β-catenin, ZO-1 and E-cadherin) and of stromal/mesenchymal markers (CD29, CD54 and vimentin). In contrast, mesothelium appeared immunonegative against WT1, CD44 and α-SMA. Nuclei are counterstained in blue with Hoechst 33342. (TIF) [file pone.0055181.s001.tif]

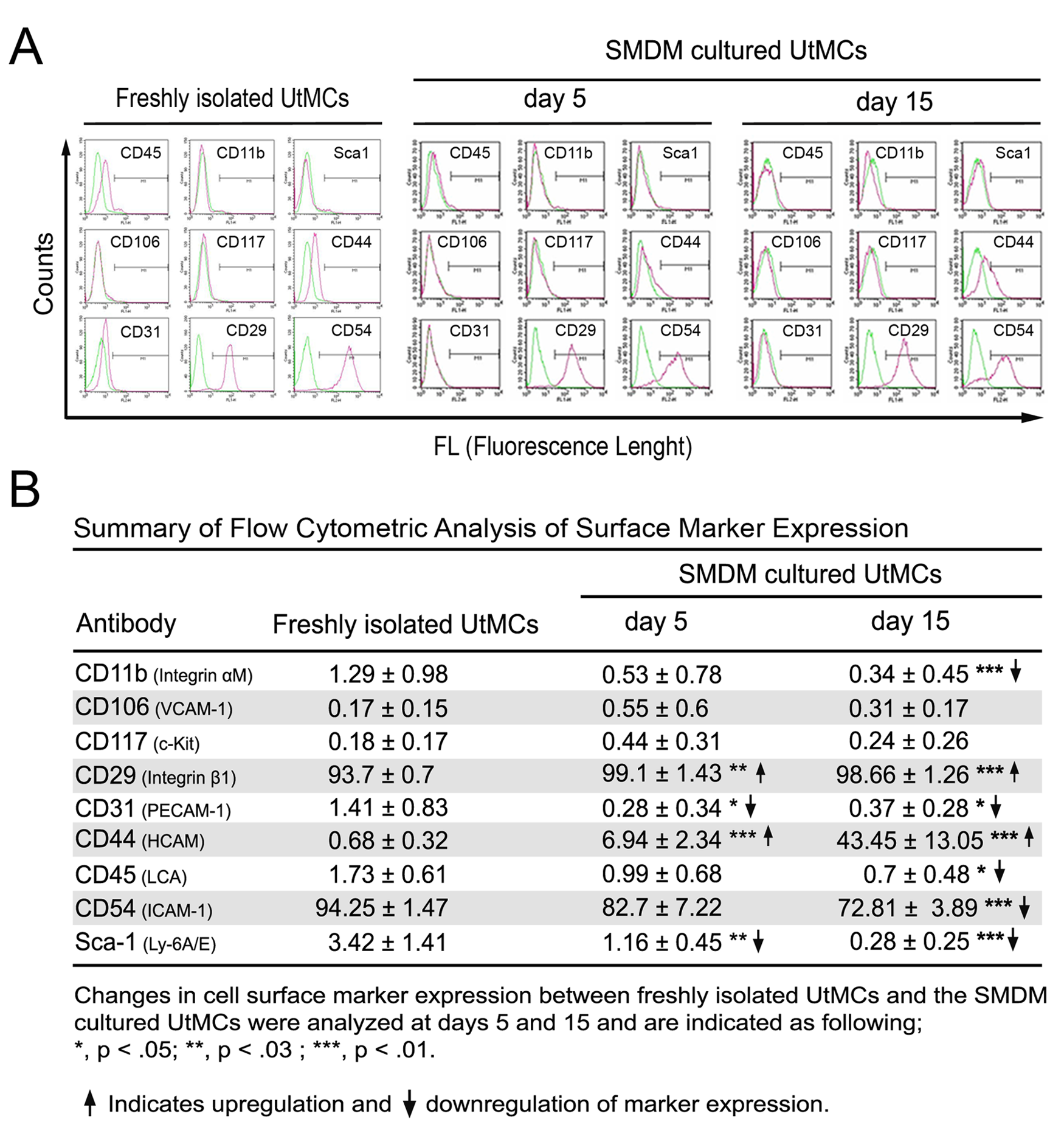

Supplement: Figure S2 — Surface marker profile analysis the freshly isolated and SMDM cultured UtMCs. (A) Freshly isolated UtMCs and UtMCs cultured in SMSM for 5 days (undergoing EMT) and 15 days (UtMCs-derived VSM-like cells) were characterized by flow cytometry. Panel shows representative histograms obtained for each marker. Pink histograms correspond to cells incubated with fluorescent-conjugated antibodies. Green histograms correspond to cells incubated with fluorescent-conjugated isotype-matched antibodies. (B) Summary results of percentages of cells marker positive ± s.d. Significant changes in cell surface marker expression were analyzed after 5 and 15 days of culture in SMDM and compared against values found in freshly isolated UtMCs; *, p<.05; **, p<.03; ***, p<.01. (TIF) [file pone.0055181.s002.tif]

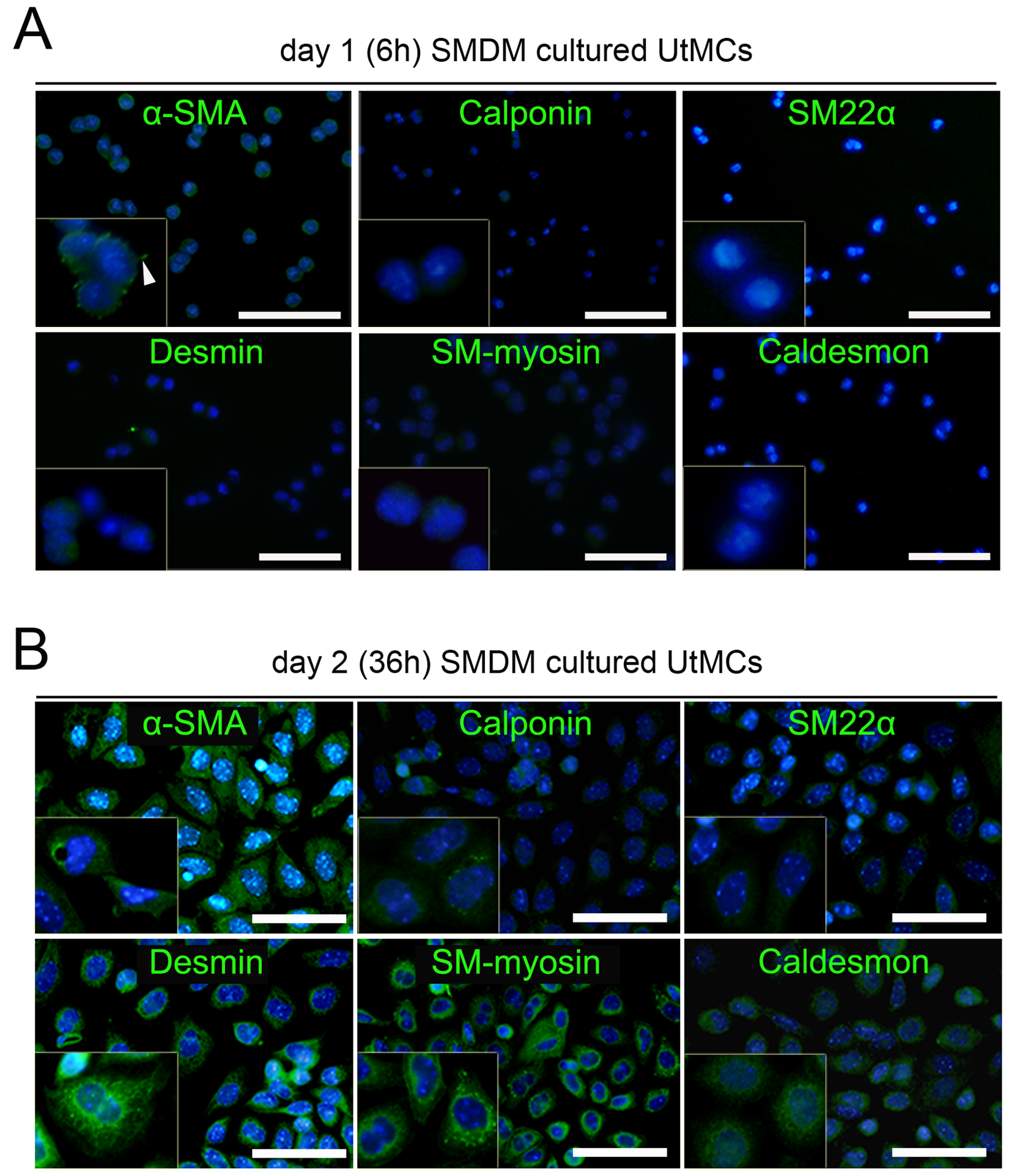

Supplement: Figure S3 — UtMCs rapidly acquire SMCs markers expression upon culture in SMDM. Upper and lower immunofluorescence panels show immunofluorescence expression levels of the SMCs markers; α-SMA, calponin, SM22α, desmin, SM-myosin and caldesmon in UtMCs cultured for 6 and 36 hours in SMDM. Nuclei are counterstained in blue with Hoechst 33342. Scale bar is 50 µm. (TIF) [file pone.0055181.s003.tif]

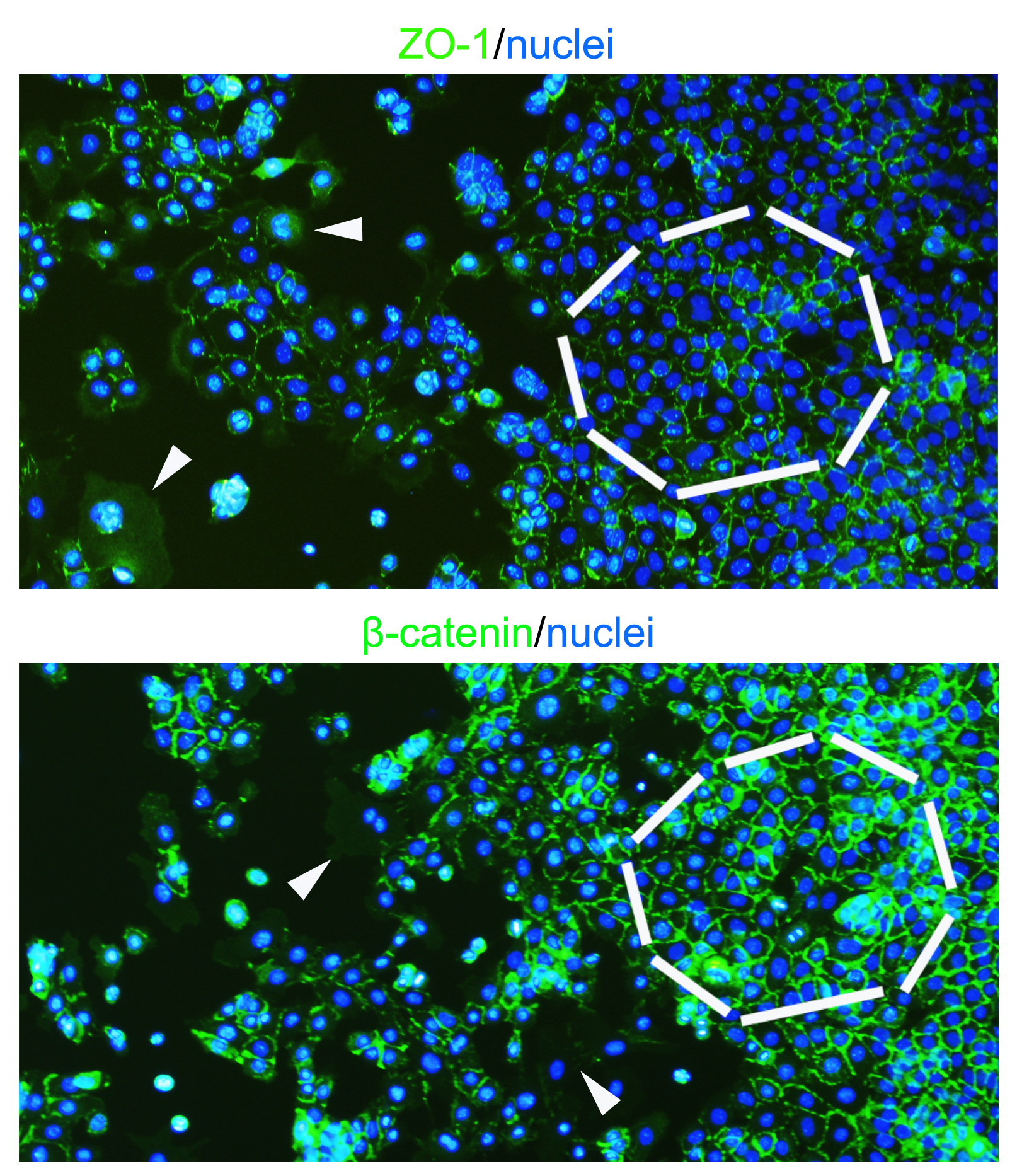

Supplement: Figure S4 — UtMCs cultured for 3 days in SMDM display epithelial type ZO-1 and β-catenin expression pattern. Immunofluorescence of ZO-1 (upper image) and β-catenin (lower image) in UtMCs cultured for 3 days in SMDM allow the visualization of UtMCs that have detached from each other (arrowheads) and that present a partial or total loss of ZO-1 and β-catenin expression. Areas with higher cell densities seeding (centre of the well) show UtMCs displaying tight cohesion and vivid intercellular immunofluorescence expression of ZO-1 and β-catenin (white dashed areas). (TIF) [file pone.0055181.s004.tif]

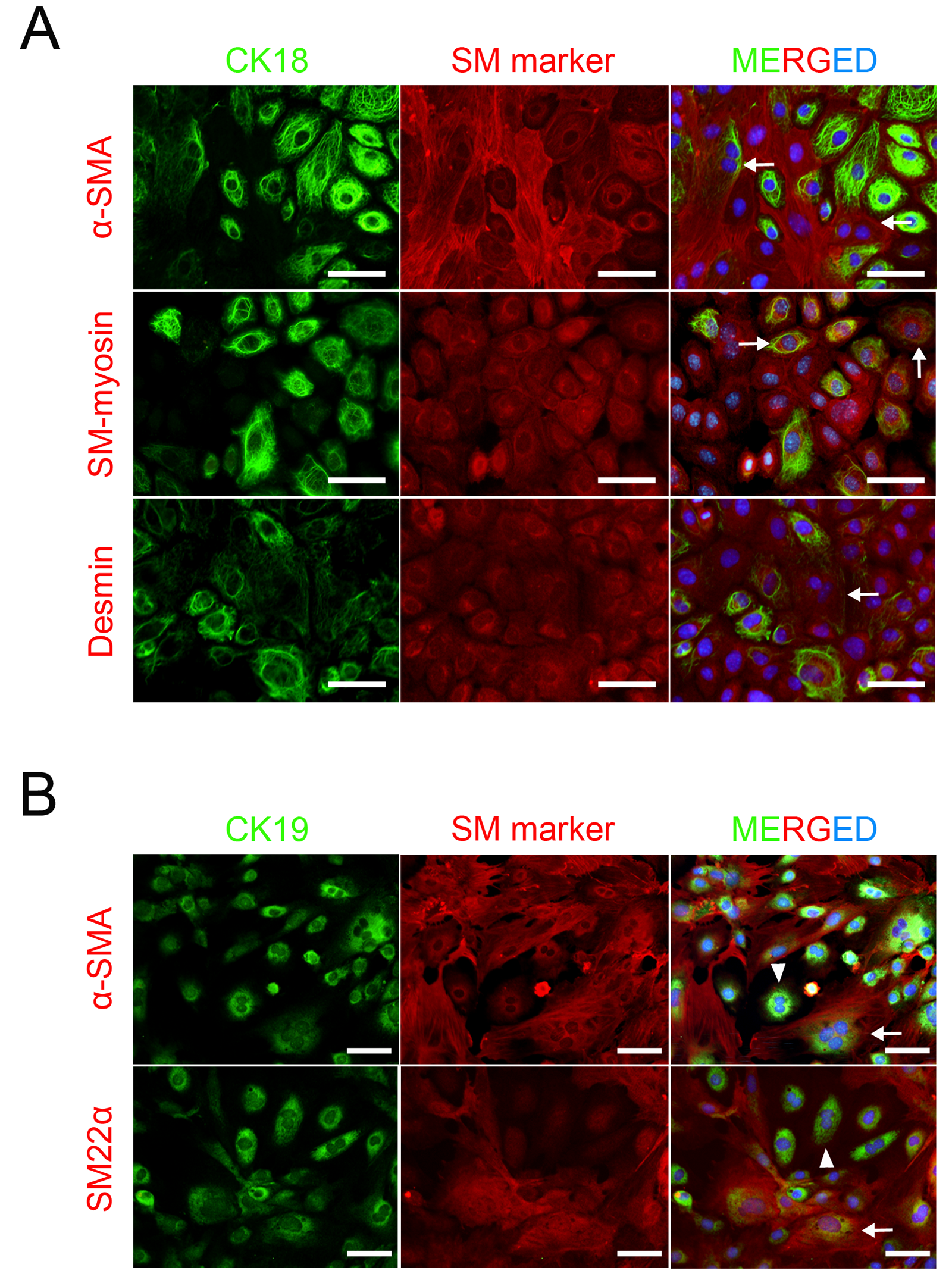

Supplement: Figure S5 — SMDM differentiating UtMCs coexpress epithelial and SM markers. (A) UtMCs cultured for 5 days in SMDM were double immunofluorescently labelled against the epithelial/mesothelial marker CK18 and the SMCs markers; α-SMA, SM-myosin and desmin. Arrows show cells co-expressing CK18 and SMCs markers. (B) UtMCs cultured for 10 days in SMDM were double immunofluorescently labelled against the epithelial/mesothelial marker CK19 and the SMCs markers; α-SMA and SM22α. Arrowheads point to UtMCs retaining a mesothelial phenotype (CK19+/α-SMA- and CK19+/SM22α-). Arrows show UtMCs-derived VSM-like cells that are detected as CK19+/α-SMA+ and CK19+/SM22α+ cells. (A–B) Nuclei are counterstained in blue with Hoechst 33342 and scale bar is 50 µm. (TIF) [file pone.0055181.s005.tif]
