# Supplementary material for: Functional Vascular Smooth Muscle-like Cells Derived from Adult Mouse Uterine Mesothelial Cells
Source: PLoS One. 2013 Feb 6;8(2):e55181. doi: 10.1371/journal.pone.0055181 (PMC3566215; doi:10.1371/journal.pone.0055181)
Supplement: Table S1 — List of primary antibodies used in this study. (DOC) [file pone.0055181.s006.doc]

**Table S1.** List of primary antibodies

| Antibody | Supplier | Reference | Clone Source Fixation IF WB | | | | |
| --- | --- | --- | --- | --- | --- | --- | --- |
| β-catenin | Cell signalling | 9581 | polyclonal | R | MeOH | 1/200 | 1/500 |
| ZO-1 | InVitrogen | 40-2200 | polyclonal | R | PFA | 1/200 | 1/500 |
| E-cadherin | BD | 610181 | 36/E-cadherin | M | MeOH | 1/50 | 1/500 |
| Cytokeratin 18 | Millipore | MAB3234 | RGE53 | M | MeOH | 1/200 | n.d |
| Cytokeratin 19 | abcam | ab15463 | polyclonal | R | PFA | 1/200 | n.d |
| WT1 | Dako | M3561 | 6F-H2 | M | MeOH | 1/100 | 1/500 |
| Nestin | Millipore | MAB353 | Rat-401 | M | PFA | 1/100 | n.d |
| βIII-tubulin | Covance | MMS-435P | TUJ1 | M | PFA | 1/400 | n.d |
| Vimentin | Millipore | AB 1620 | polyclonal | G | PFA | 1/200 | n.d |
| Desmin | Sigma | D 8281 | antisera | R | PFA | 1/50 | n.d |
| α-SMA | Sigma | A 5228 | 1A4 | M | MeOH | 1/300 | 1/1000 |
| Calponin | Sigma | C 2687 | hCP | M | PFA | 1/100 | n.d |
| SM22α | abcam | ab28811 | 1B8 | M | PFA | 1/200 | n.d |
| SM-myosin | Sigma | M 7648 | antisera | R | PFA | 1/50 | n.d |
| Caldesmon | Sigma | C 4562 | hHCD | M | PFA | 1/100 | n.d |
| Smoothelin-B | SCBT | Sc-28562 | polyclonal | R | PFA | 1/200 | 1/500 |
| mAChR M3 | SCBT | sc-9108 | H-210 | R | PFA | 1/200 | n.d |
| mAChR M2 | SCBT | sc-9107 | H-170 | R | PFA | 1/200 | n.d |
| Gata-4 | SCBT | sc-25310 | G-4 | M | MeOH | 1/100 | 1/300 |
| cTnT | abcam | ab10214 | 1 F-11 | M | PFA | 1/200 | 1/500 |
| α-actinin sarcomeric | Sigma | A 7811 | EA-53 | M | PFA | 1/200 | 1/500 |
| ANF | Millipore | AB5490 | polyclonal | R | PFA | 1/100 | n.d |
| CD68 | Serotec | MCA1957GA | FA-11 | R | PFA | 1/200 | n.d |
| F4/80 | Serotec | MCA497GA | Cl:A3-1 | R | PFA | 1/100 | n.d |
| PDGFR-β | SCBT | Sc-432 | polyclonal | R | PFA | 1/100 | 1/500 |
| Isl1 | Chemicon | AB5754 | polyclonal | R | PFA | 1/100 | 1/500 |
| Sox2 | RD | MAB2018 | 245610 | M | PFA | 1/100 | 1/500 |
| Nanog | abcam | ab21603 | polyclonal | R | PFA | 1/200 | 1/500 |
| Oct-3/4 | BD | 084720 | 40 | M | PFA | 1/100 | 1/500 |
| GAPDH | Cell signalling | 2118 | polyclonal | R | PFA | n.d | 1/1000 |
| Abbreviations: ANF; atrial natriuretic factor; cTnT, cardiac troponin T; mAChR, muscarinic acetylcholine receptors; GAPDH, glyceraldehyde 3-phosphate dehydrogenase; MHC, myosin heavy chain; PDGFR-β, platelet derived growth factor receptor type β; WT1, Wilm´s tumor protein 1; ZO-1, zona occludens-1. BD, Becton Dickinson; SCBT, Santa Cruz Biotechnology; RD, R&D Systems. R, rabbit, M, mouse, G, goat. PFA, paraformaldehyde; MeOH, methanol. n.d, not done. | | | | | | | |
