# Supplementary material for: Functional Vascular Smooth Muscle-like Cells Derived from Adult Mouse Uterine Mesothelial Cells
Source: PLoS One. 2013 Feb 6;8(2):e55181. doi: 10.1371/journal.pone.0055181 (PMC3566215; doi:10.1371/journal.pone.0055181)
Supplement: Table S2 — List of secondary antibodies used in this study. (DOC) [file pone.0055181.s007.doc]

**Table S2**. List of secondary antibodies

| Antibody | Supplier | Reference | Label | Species Dilution | |
| --- | --- | --- | --- | --- | --- |
| **Mouse IgG** | InVitrogen | A11029 | AF 488 nm | Goat | 1/300 |
| **Mouse IgG** | InVitrogen | A11032 | AF 594 nm | Goat | 1/300 |
| **Rabbit IgG** | InVitrogen | A11034 | AF 488 nm | Goat | 1/300 |
| **Rabbit IgG** | InVitrogen | A11037 | AF 594 nm | Goat | 1/300 |
| **Goat IgG** | InVitrogen | A11057 | AF 568 nm | Donkey | 1/300 |
| **Goat IgG** | InVitrogen | A11078 | AF 488 nm | Rabbit | 1/300 |
| **Rat IgG** | InVitrogen | A11077 | AF 568 nm | Goat | 1/300 |
| **Mouse IgG** | Sigma-Aldrich | A9044 | HRP | Rabbit | 1/20000 |
| **Rabbit IgG** | Sigma-Aldrich | A9169 | HRP | Goat | 1/20000 |
| **Goat IgG** | Jackson L. | 705-036-147 | HRP | Donkey | 1/20000 |
