# Supplementary material for: Functional Vascular Smooth Muscle-like Cells Derived from Adult Mouse Uterine Mesothelial Cells
Source: PLoS One. 2013 Feb 6;8(2):e55181. doi: 10.1371/journal.pone.0055181 (PMC3566215; doi:10.1371/journal.pone.0055181)
Supplement: Table S3 — List of fluorescent conjugated antibodies used in this study. (DOCX) [file pone.0055181.s008.docx]

**Table S3**. List of fluorescent conjugated antibodies

| Antibody | Supplier | | Reference | Label | Species Dilution | |
| --- | --- | --- | --- | --- | --- | --- |
| **CD11b** (Integrin αM) | | BD | 553310 | FITC | rat | 1/100 |
| **CD106** (VCAM-1) | | BD | 553332 | FITC | rat | 1/100 |
| **CD117** (c-Kit) | | BD | 553354 | FITC | rat | 1/100 |
| **CD29** (Integrin β1) | | BD | 555005 | FITC | hamster | 1/100 |
| **CD31** (PECAM-1) | | BD | 553373 | R-PE | rat | 1/100 |
| **CD44** (HCAM) | | BD | 553133 | FITC | rat | 1/100 |
| **CD45** (LCA) | | BD | 553080 | FITC | rat | 1/100 |
| **CD54** (ICAM-1) | | BD | 553253 | R-PE | hamster | 1/100 |
| **Sca-1** (Ly-6A/E) | | BD | 553335 | FITC | rat | 1/100 |
| **Rat IgG2b,k** | | BD | 553988 | FITC | - | 1/100 |
| **Rat IgG2a** | | BD | 553930 | R-PE | - | 1/100 |
| **Hamster IgG1,K** | | BD | 553972 | R-PE | - | 1/100 |
| **Hamster IgM** | | BD | 553960 | FITC | - | 1/100 |
| Abbreviations: **HCAM**, homing cell adhesion molecule; **ICAM-1**, intercellular cell adhesion molecule 1; **LCA**, leukocyte common antigen; **PECAM-1**, platelet endothelial cell adhesion molecule 1; **VCAM-1**, vascular cell adhesion molecule 1; **BD**, Becton Dickinson; **FITC**, fluorescein isothiocyanate; **R-PE**; R-phycoerythrin. | | | | | | |
