# Supplementary material for: Functional Vascular Smooth Muscle-like Cells Derived from Adult Mouse Uterine Mesothelial Cells
Source: PLoS One. 2013 Feb 6;8(2):e55181. doi: 10.1371/journal.pone.0055181 (PMC3566215; doi:10.1371/journal.pone.0055181)
Supplement: Table S4 — List of primers used for Reverse Transcriptase-Polymerase Chain Reaction. (DOCX) [file pone.0055181.s009.docx]

**Table S4.** Primers used for Reverse Transcriptase-Polymerase Chain Reaction

| *Gene* | *Forward* | *Reverse* | *pb* | *TºC* |
| --- | --- | --- | --- | --- |
| **E-cadherin** | CCTGTCTTCAACCCAAGC | ATTTCCTGACCCACACCAAA | 398 | 58 |
| **CK18** | CAAGTCTGCCGAAATCAGGGAC | TCCAAGTTGATGTTCTGGTTTT | 111 | 60 |
| **WT1** | CCAGTGTAAAACTTGTCAGCGA | TGGGATGCTGCACTGTCT | 234 | 55 |
| **Twist** | CCAGAGAAGGAGAAAATGGACAGTC | AAAAAGTGGGGTGGGGGGACACAAAC | 259 | 60 |
| **Snail** | TCCAAACCCACTCGGATGTGAAGA | TTGGTGCTTGTGGAGCAAGGACAT | 86 | 60 |
| **Slug** | CACATTCGAACCCACACATTGCCT | TGTGCCCTCAGGTTTGATCTGTCT | 164 | 60 |
| **α-SMA** | ACGGCCGCCTCCTCTTCCTC | GCCCAGCTTCGTCGTATTCC | 415 | 60 |
| **SM22α** | GCAGTCCAAAATTGAGAAGA | CTGTTGCTGCCCATTTGAAG | 507 | 55 |
| **Caldesmon** | AGAGATCGAAAGGAGAAGGG | CTATCTTGAGAGACGAGCCT | 122 | 60 |
| **Calponin** | acatcattggactgcagatg | CAAAGATCTGCCGCTTGGTG | 204 | 60 |
| **SM-MHC** | CATGGACCCGCTAAATGACA | CAATGCGGTCCACATCCTTC | 92 | 55 |
| **Desmin** | TCGTATTGACCTGGAGCGCAGAAT | ATGTTCTTAGCCGCGATGGTCTCA | 204 | 60 |
| **Smtn-A/B** | CGTTTACCATTGAGATCAAGGATGG | TCGGTTACCAGGGGAGGCATGGCTG | 236 | 60 |
| **Smtn-B** | TCAGAGGCTTCTCCAACACTAAGAG | TTGGCTCTCGATTTGGGGTTGGTTG | 108 | 60 |
| **Gata-4** | TCTCACTATGGGCACAGCAG | GCGATGTCTGAGTGACAGGA | 136 | 60 |
| **Nkx2.5** | CAAGTGCTCTCCTGCTTTCC | GGCTTTGTCCAGCTCCACT | 136 | 55 |
| **Isl1** | CGGAGAGACATGATGGTGGTT | GGCTGATCTATGTCGCTTTGC | 109 | 55 |
| **Tbx5** | GGAGCCTGATTCCAAAGACA | TTCAGCCACAGTTCACGTTC | 153 | 55 |
| **C-kit** | TCATCGAGTGTGATGGGAAA | GGTGACTTGTTTCAGGCACA | 222 | 55 |
| **Hrt1** | AGTGAGCTGGACGAGACCAT | CTGGGTACCAGCCTTCTCAG | 197 | 55 |
| **BMP2** | CTCCACAAACGAGAAAAGCG | CATGCCTTAGGGATTTTGGA | 254 | 60 |
| **BMP4** | CTCCCAAGAATCATGGACTG | AAAGCAGAGCTCTCACTGGT | 468 | 55 |
| **Flk1** | GGCGGTGGTGACAGTATCTT | CTCGGTGATGTACACGATGC | 189 | 60 |
| **SRF** | TCTCAGGCACCATCCACCAT | CCCAGCTTGCTGCCCTATCAC | 156 | 60 |
| **MYOCD** | TTCTGGGTTGTTAGCTGCTGTCCT | ATGTGCATAGTAACCAGGCTGGCA | 344 | 58 |
| **ACTC1** | CTGGATTCTGGCGATGGTGTA | CGGACAATTTCACGTTCAGCA | 173 | 55 |
| **cTnT** | CAGAGGAGGCCAACGTAGAAG | CTCCATCGGGGATCTTGGGT | 138 | 58 |
| **MHC-α** | GAGATTTCTCCAACCCAG | TCTGACTTTCGGAGGTACT | 225 | 55 |
| **MHC-β** | CTACAGGCCTGGGCTTACCT | TCTCCTTCTCAGACTTCCGC | 126 | 55 |
| **MLC2a** | TCAGCTGCATTGACCAGAAC | AAGACGGTGAAGTTGATGGG | 148 | 55 |
| **ANF** | GGAGCCTACGAAGATCCAGC | TCCAATCCTGTCAATCCTACCC | 50 | 55 |
| **Cx43** | CTGTACTTGGCTCACGTGTTCTAT | CGTGGGAGTTGGAGATGGTGC | 724 | 58 |
| **SERCA2** | CTGTGGAGACCCTTGGTTGT | CAGAGCACAGATGGTGGCTA | 245 | 58 |
| **Sox2** | CACAGATGCAACCGATGCA | GGTGCCCTGCTGCGAGTA | 122 | 55 |
| **Oct-3/4** | GGCGTTCTCTTTGGAAAGGT | TCTCATTGTTGTCGGCTTCCT | 133 | 55 |
| **Nanog** | AGGGTCTGCTACTGAGATGCTC | CAACCACTGGTTTTTCTGCCACC | 364 | 60 |
| **β-actin** | TGGGAATGGGTCAGAAGGAC | TGAAGCTGTAGCCACGCTCG | 468 | 60 |

Abbreviations: **ACTC1**, alpha-cardiac actin 1; **ANF**, atrial natriuretic factor; **α-SMA**, alpha smooth muscle actin; **BMP2**, bone morphogenetic protein 2; **BMP4**, bone morphogenetic protein 4; **CK18**, cytokeratin 18; **cTnT**, cardiac troponin T; **cTnI**, cardiac troponin I; **Cx43**, connexin-43; Flk1 (Fetal Liver Kinase 1), also known as vascular endothelial growth factor receptor 2 (VEGFR-2); **Hrt1**, heart protein 1; **Isl1**, ISL LIM homeobox 1; **MHC-α**, myosin heavy chain alpha; **MLC2a**, myosin light chain 2a; **MYOCD**, myocardin; **SM-MHC**, smooth muscle myosin heavy chain; **SERCA2**, sarco/endoplasmic reticulum Ca2+-ATPase; **Smtn-A/B**, smoothelin-A/B; **Smtn-B**, smoothelin-B; **SRF**, serum response factor; **Tbx5**, T-box 5; **Sox2**, SRY-box 2; **WT1**, Wilm´s tumor protein 1.
